# Benefits of group sequential design and sample size re-estimation for randomised controlled trials evaluating the prevention of ventilator-associated pneumonia: a simulation study informed by real world data

**DOI:** 10.1186/s12874-025-02681-4

**Published:** 2025-11-12

**Authors:** Holly Jackson, Julien Sauser, C. H. van Werkhoven, Stephan Harbarth, Marlieke E.A. de Kraker

**Affiliations:** 1 Infection Control Program, Faculty of Medicine, Geneva University Hospitals, World Health Organization Collaborating Center, Geneva, Switzerland; 2 Clinical Research Center, University Hospital of Lausanne and University of Lausanne, Lausanne, Switzerland; 3 Julius Center for Health Sciences and Primary Care, University Medical Center Utrecht, Utrecht University, Utrecht, the Netherlands

**Keywords:** Randomised clinical trial, Adaptive clinical trial design, Group sequential design, Sample size re-estimation, Ventilator-associated pneumonia

## Abstract

**Background:**

Ventilator-associated pneumonia (VAP) is an important healthcare acquired infection, which is associated with high morbidity and mortality. Conducting conventional randomised controlled trials (RCTs) on VAP prevention is often challenging, due to low numbers of eligible patients and events per site, especially for pathogen-specific interventions. We explored how group sequential designs (GSD) and sample size re-estimation (SSR) trial designs could improve RCT efficiency in simulated superiority trials to prevent VAP.

**Methods:**

Simulations were informed using data from the prospective observational Hospital Network Study – Preparation for a Randomised Evaluation of anti-Pneumonia Strategies (HONEST-PREPS). We tested the impact of different GSD and SSR designs on expected sample size (considering early stopping) and maximum sample size (no early stopping). We varied the type of stopping boundary, number and timepoint of interim analyses, and assumed and true prevention effect. We applied time-to-event analyses, with effect estimates expressed as hazard ratios, for the primary endpoint.

**Results:**

The estimated 28-day cumulative incidence of VAP in HONEST-PREPS was 15.5%. For a 30% reduction in VAP (hazard ratio of 0.68), a standard RCT (power 80%) would require a sample size of 1291 patients. For GSD, Pocock boundaries result in a smaller expected sample size (E[N] = 1128), but a larger maximum sample size (max(N) = 1578) than O’Brien Fleming boundaries (E[N] = 1170 and max(N) = 1389), when utilising the optimal placement of a single interim analysis, 48% and 64% of the maximum number of events for Pocock and O’Brien Fleming boundaries, respectively. SSR is more efficient compared to GSD when the incorrect prevention effect is initially used to plan the trial, as it maintains a power closer to the pre-specified desired power without substantial impact on the expected sample size.

**Conclusions:**

GSD and SSR are effective adaptive designs, preferable to fixed RCTs in a superiority trial comparing the effectiveness of an investigational intervention with a standard of care in preventing VAP among critically ill, ventilated patients. They can reduce the expected sample size between 9% and 12% and should be considered at the trial design stage.

**Supplementary Information:**

The online version contains supplementary material available at 10.1186/s12874-025-02681-4.

## Introduction

Emerging infectious diseases and antimicrobial resistance are growing threats, which require efficient approaches to evaluate novel preventive and therapeutic strategies. Unfortunately, the conventional randomised controlled trial (RCT) is challenging in these domains, often too few patients can be timely enrolled, resulting in an underpowered RCT and a waste of resources [1]. To increase benefit to patients, more efficient trials, such as adaptive trial designs, are needed. Although the COVID-19 pandemic has increased uptake, adaptive elements are still not routinely considered, especially in the field of infectious diseases. This is partly associated with their complexity and lack of understanding by clinicians, funders, and regulators [2].

Adaptive trial designs are more flexible than the conventional RCT, as they allow trial modifications based on pre-specified decision rules, allowing more efficient use of the collected data [3–5]. Group sequential designs (GSD) and sample size re-estimation (SSR) can be incorporated relatively easily within an RCT. In GSDs, pre-planned interim analyses (IAs) allow a trial to end early for efficacy or futility [6]. When applying a GSD: the number of IAs, their timing, and the stopping boundaries need to be pre-specified [7]. Although there exist investigations in the statistical literature to determine their optimal choices, particularly when the patient outcome is binary or normally distributed [8], there is little empirical data showing what would be optimal choices for time to event outcomes when considering the distribution of events and the accrual intensity, as these can vary depending on the particular case.

SSR is an extension to the GSD, where the sample size of the trial may be re-calculated at IA(s) [9], i.e. there is no fixed maximum sample size. Decisions about changing the sample size depend on pre-specified criteria, and at each IA in theory one of five decisions can be made; stop the RCT for evidence of efficacy, continue the RCT with a decrease in the planned sample size to ensure adequate power to detect a larger than anticipated effect size, continue as planned, continue with an increased sample size to ensure adequate power to detect a smaller than anticipated effect size, or stop the trial for futility if the difference between treatment arms is small and/or the sample size required to find a difference would be too large [10]. This type of design can be particularly helpful when there is uncertainty about the effect size of a novel intervention or uncertainty in the distribution of the outcome in the control arm, as the originally calculated sample size may be incorrect [11]. In addition to the parameters needed for a GSD, the SSR design also needs to pre-specify the timing of SSR and whether and to what extent the sample size can decrease or increase.

For adaptive designs, it is important that the endpoint is recorded quickly, relative to the trial recruitment rate, to allow the IA to make impactful decisions on the trial design [12]. The longer the delay in observing the outcome, relative to recruitment rate, the less efficient the design becomes. Therefore, when these designs are being considered, exploring the best time placement for IA(s) for the particular case (dependent on recruitment rate and time to event) is imperative. Recently, there have been several RCTs looking at the prevention of ventilator-associated pneumonia (VAP) [13–17]. GSD and/or SSR could be beneficial in this setting as VAP normally occurs relatively quickly after the start of the at-risk period (under invasive mechanical ventilation (IMV) for at least 48 h) with a median time to VAP diagnosis of less than 10 days [13–17].

The objective of this manuscript is to evaluate the feasibility and added value of GSD and SSR in RCTs focused on prevention of VAP, informed by real world data about VAP in the intensive care unit (ICU).

## Methods

### Data

The Hospital Network Study – Preparation for a Randomised Evaluation of anti-Pneumonia Strategies (HONEST-PREPS), provided the required data. It was a prospective cohort study enrolling patients ≥ 18 years old admitted to the ICU at risk of hospital-acquired pneumonia or VAP (expected or documented length of hospitalisation of more than 48 h) in 12 ICUs across 6 European countries from 2020 to 2022 (ClinicalTrials.gov ID: NCT05060718) [18–20]. All patients, or their legal representatives, had to provide informed consent for participation. In HONEST-PREPS, VAP was defined according to Food and Drug Administration (FDA) criteria [21]. For this current study, patients were excluded if they were not ventilated for at least 48 h during their ICU stay.

## Simulation study

### Setting

We compare the GSD and SSR designs to a conventional fixed RCT, in a parallel group, two-arm, superiority trial to assess the effectiveness of a new investigational intervention (Inv) compared to the standard of care (SoC) to reduce the incidence of VAP, among patients under IMV for at least 48 h. We assume a 1:1 allocation ratio. The performance measures of interest are the expected and maximum sample size, see below. The data from HONEST-PREPS was used to find the average accrual intensity, and fit models for VAP incidence, and distribution of VAP onset time, see the code in additional material 2. The values utilised within the simulation study were calculated from these fitted models.

### Design choices

Simulations are separated into three individual parts. We first compared a fixed RCT to a GSD with O’Brien Fleming (OBF) and Pocock alpha- and non-binding beta-spending functions [22], with a single IA placed at various timings throughout the study. We chose non-binding beta-spending functions as they tend to be more common in clinical trial practice [23] and we assume early stopping at the IA for futility, if these boundaries are crossed throughout all our investigations. Pocock boundaries are narrower than OBF boundaries (Figure SM1), which means that the trial is more likely to stop at IAs performed at the same time point. However, a trial utilising the OBF boundaries is more likely to reject the null hypothesis at the final analysis. Secondly, the impact of the number of IAs (one to nine) used in a GSD with OBF boundaries was investigated. Here, we assumed the IAs would be equally spaced throughout the trial. Finally, we compared a fixed RCT to a GSD, SSR only allowing an increase in sample size (SSR-increase) and SSR allowing an increase or decrease in sample size (SSR-both) using OBF boundaries, with one IA, in two settings. Optimal timing of the IA was selected, assessed by finding the minimum number of expected events in a GSD.

In the first comparison, for GSD and fixed RCT designs, we used the “true” prevention effect to calculate their sample sizes. For both SSR designs, we started with a smaller study, powered to detect a realistic prevention effect – that was larger than the smallest clinically meaningful, prevention effect (underpowered), this was then increased appropriately at the IA [24]. We used an absolute prevention effect which was 5% larger than the true prevention effect in the investigational intervention arm, to calculate the initial sample size and then re-calculated the sample size, using the observed prevention effect at IA.

In the second comparison, we conceptualised a frequently occurring design dilemma when the expected prevention effect is larger than the minimal clinically relevant prevention effect. Investigators can initially base the sample size on the relevant effect size, resulting in a larger trial (“pessimistic” design), or on the expected effect size, resulting in a smaller trial that is underpowered if the true effect is equal to the relevant effect (“greedy” design). We contrasted the sample sizes of a fixed RCT, GSD, SSR-increase and SSR-both while exploring the impact of using an initial prevention effect to calculate the sample size of the trial, which was lower and higher than the “true” prevention effect for all four designs. Where the initial prevention effect is higher, this can be conceptualised as a “greedy” design, and the sample size should be re-estimated at IA within the SSR-design considering the smaller “true” effect. When the initial prevention effect is smaller, this would be a “pessimistic” design, and we re-estimate the sample size at IA within the SSR-design considering a larger “true” effect.

Furthermore, we applied the following assumptions to all trial designs: one sided type I error of 2.5%, patients are followed for a maximum of 1 month from enrolment (which is long enough to determine day 28 VAP rate), there are no dropouts, power of 80% (main text) and 90% (additional material 1). The GSDs utilised formal efficacy and non-binding futility stopping boundaries. The SSR designs utilised formal efficacy stopping boundaries, however, we could stop these trials for futility if the increase in sample size at interim analysis was too high or if the prevention effect observed at IA favoured the SoC, described in more detail below.

### Sample size calculation

We used time-to-event methodology to compute the sample sizes assuming proportional hazards. The use of time to VAP diagnosis as an endpoint is preferred over a single measure of difference in risks between the treatment arms, as it also includes the time taken for VAP to occur and the potential variation in follow-up time [25], as in the trial [16]. Therefore, we used hazard ratios (HRs) to calculate the expected and maximum number of events needed for each trial design.

For a fixed RCT, Eq. 1 can be used to find the total number of events, $\:n$, which produces a trial with power $\:(1-\beta\:)$ and maintains one-sided type I error, α, where $\:{P}_{k}$ is the proportion of participants allocated to each treatment arm $\:k$: SoC or investigational [26].

1 $$\:n=\frac{{({Z}_{\left(1-\alpha\:\right)}+{Z}_{(1-\beta\:)})}^{2}}{{P}_{Inv}\:\bullet\:\:{P}_{SoC}\:\bullet\:\:{\text{ln}\left(HR\right)}^{2}}$$

Then the sample size is defined as the ratio between the number of events (i.e. the number of patients who develop VAP) and the probability of observing an event during the trial [25]. To compute the denominator, we assume a piecewise exponential distribution for VAP cumulative incidence, based on the HONEST-PREPS data. All analyses described below were performed using the statistical program ‘R’ version 4.4.2 [27], using the package ‘rpact’ [28] and the code is available in additional material 2.

### Scenarios

We investigated these trial designs by utilising a series of five simulated parallel group, two-arm, superiority interventional trials. We explored a range of risk differences (based around a 32% reduction in 28-day VAP cumulative incidence as found in SAATELITE [14] and AMIKINHAL [16]). We derived the HR per scenario, by utilising the risk difference and the 28-day VAP cumulative incidence in the SoC arm estimated from the HONEST-PREPS data, Table 1.

**Table 1:** 28-day cumulative incidence of ventilator-associated pneumonia (VAP) in the standard of care arm (SoC) and the investigational intervention (Inv) per scenario

Scenario	Cumulative incidence of VAP in SoC	% Reduction in Inv	Cumulative incidence of VAP in Inv	Hazard Ratio
**1**	15.5%	20%	12.4%	0.79
**2**	15.5%	25%	11.6%	0.73
**3**	15.5%	30%	10.8%	0.68
**4**	15.5%	35%	10.1%	0.63
**5**	15.5%	40%	9.3%	0.58

### Performance measures

We report both the expected and maximum number of events, for each trial design, within each scenario explored. As the fixed RCT includes no IA, these two measures are equal.

The expected number of events, $\:E\left[n\right]$, within a trial is defined as the average number of events (number of patients who develop VAP), given the probability of stopping the trial at each IA or continuing to final analysis [7].

2 $$\:E\left[n\right]={\sum\:}_{i=1}^{I}{p}_{i}\bullet\:{n}_{i}$$

Here $\:{p}_{i}$ is the probability of stopping the trial after the $\:i$th analysis for all $\:i=\{1,\:2,\:\dots\:,\:I\}$, where $\:{\sum\:}_{i=1}^{I}{p}_{i}=1$ and $\:I$ is the total number of planned IAs $\:+1$. Furthermore, $\:{n}_{i}$ is the number of events (across the SoC and investigational intervention arms) in all stages, 1 to $\:i$, of the trial for all $\:i=\{1,\:2,\:\dots\:,\:I\}$.

In the GSD, the maximum number of events is the total number of events across both treatment arms and all stages of the trial, assuming the trial does not stop at any IAs.

In our setting, we focus on SSR using the inverse normal combination test design, with only one IA. At the IA, the trial can either (1) stop for efficacy using the formal OBF alpha-spending boundaries (2) stop for futility if the observed HR ≥ 1 (3) proceed to the second stage, where the number of events needed is recalculated based on the observed HR from stage 1. In each scenario we must assign a *total* maximum number of events which could be observed in the trial. This is chosen via an optimisation function utilising a simulation approach and an optimisation effect size, to ensure the conditional power equals the desired power over all simulations and we limit it to be at most double what is needed in a fixed RCT using the assumed HR. The optimisation effect size is set equal to the clinically relevant effect size with the “greedy” design, and to the expected effect size with the “pessimistic” design (see above). Across the simulations the SSR design can increase the maximum number of events in the trial up to the optimised total maximum number of events. The largest maximum number of events across all simulations is known as the total maximum number of events. The average of these maximum number of events is known as the *mean* maximum number of events and is used as a performance measure for SSR. If the conditional power will not reach the desired power without increasing the total maximum number of events past the limit, then we stop the trial at the IA for futility. Here, the term ‘conditional power’ refers to the proportion of the simulations where the null hypothesis is correctly rejected at any stage (interim or final analysis) given the data observed at the interim analysis, and the simulation concludes, correctly, the superiority of the investigational intervention [29].

## Results

HONEST-PREPS recruited a total of 1,096 patients at risk of VAP, i.e. under IMV for at least 48 h, of which 170 patients (cumulative incidence 15.5%) developed VAP by day 28. Median time between start of IMV (+ 48 h) and VAP onset was 3 days (IQR: 1–6). Average accrual intensity was 47 patients/month. There were 5 ventilated patients who were lost to follow-up and censored at their last follow-up date.

### Fixed RCT

In a fixed RCT, based on an effect size (HR) of 0.58–0.79, we need 106–566 events, and 747–2710 patients to show superiority of the investigational intervention (Table SM1 in the additional material 1).

### Group sequential design

**Fig. 1:**
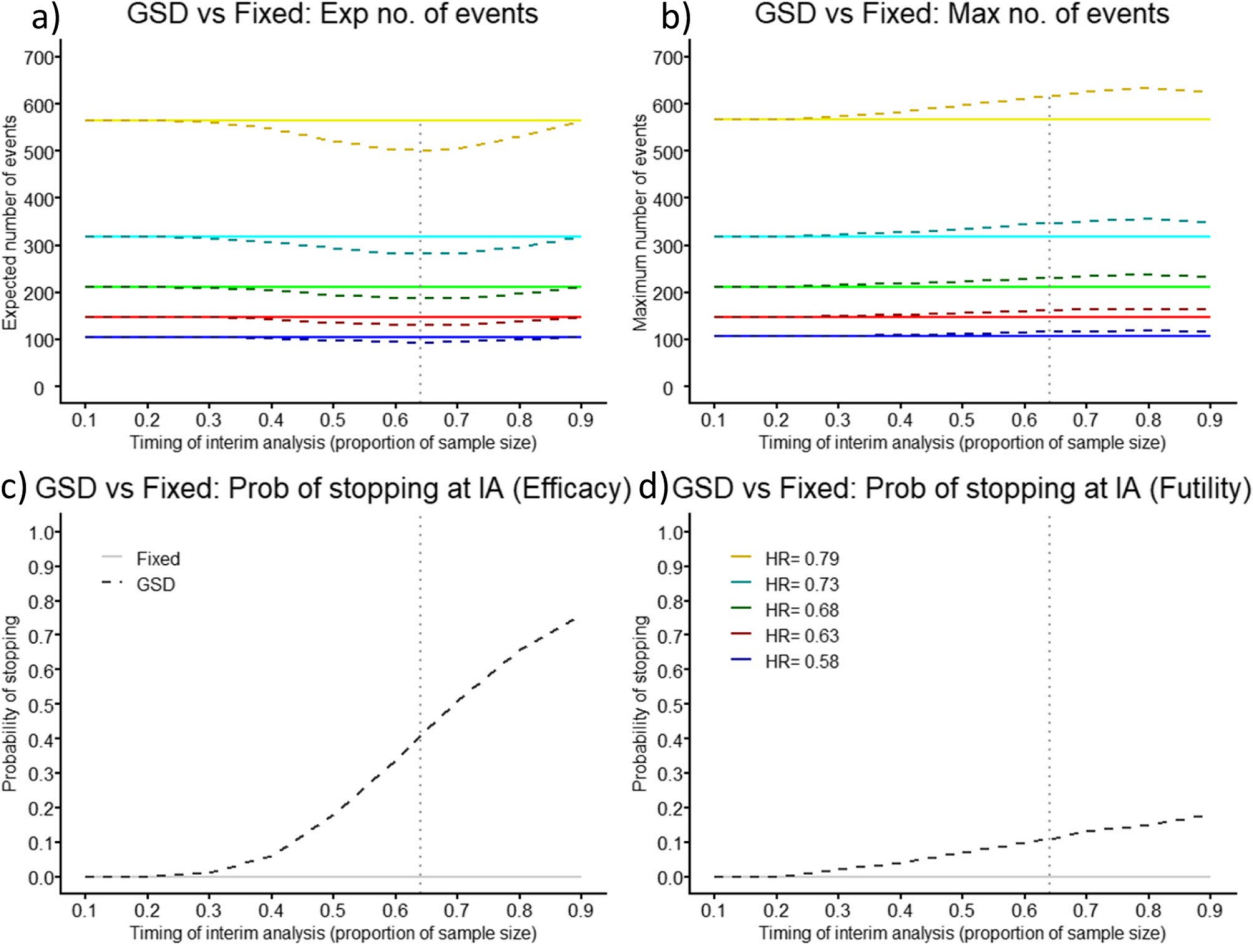
Comparison of Group Sequential Design (GSD) with O’Brien-Fleming boundaries (dashed) against Fixed Randomised Controlled Trial (solid) as timing of interim analysis (IA) placement changes showing (**a**) expected (Exp) number of events, (**b**) maximum (Max) number of events, (**c**) probability (Prob) of stopping for efficacy at the IA, and (**d**) Prob of stopping for futility at the IA, for five different prevention effects, for power of 80%

The GSD with one IA, utilising OBF boundaries to allow early stopping at the IA for efficacy and futility, produces a smaller expected number of events than the fixed RCT. When powering a trial at 80%, the optimal placement of the single IA is at 64% of the maximum number of events which is calculated for the GSD trial. This gives 41% and 11% probability of stopping the RCT at the IA for efficacy and futility, respectively, which is true across prevention effects (Figs. 1c-d). This results in an expected 502 events and 2470 patients, saving 64 events (11.3%) and 240 patients (8.9%), compared to the fixed RCT (566 events and 2710 patients) in scenario 1, when prevention effect is low (HR = 0.79). The distribution of required events is shown in Figure SM2a in the additional material 1. For scenario 5 (HR = 0.58), high prevention effect, there is a smaller absolute but comparable relative saving of 12 events (11.3%; Fixed = 106 vs. GSD = 94) and 71 patients (9.5%; Fixed = 747 vs. GSD = 676) (Fig. 1a, Table SM1 in the additional material 1). The distribution of required events is shown in Figure SM2b in the additional material 1.

The savings in expected sample size comes at the cost of an increase in the maximum sample size. If the trial does not stop at the optimal IA, it would require 51 more events (9.0%) and 186 more patients (6.9%), than in the fixed design (scenario 1), or 10 additional events (9.4%) and 60 additional patients (8.0%) (scenario 5) (Fig. 1b, Table SM1 in the additional material 1).

When the power is 90%, optimal IA placement, relative gains in expected sample size and relative increase of maximum sample size are similar (Figure SM3, Table SM2 in the additional material 1).

**Fig. 2:**
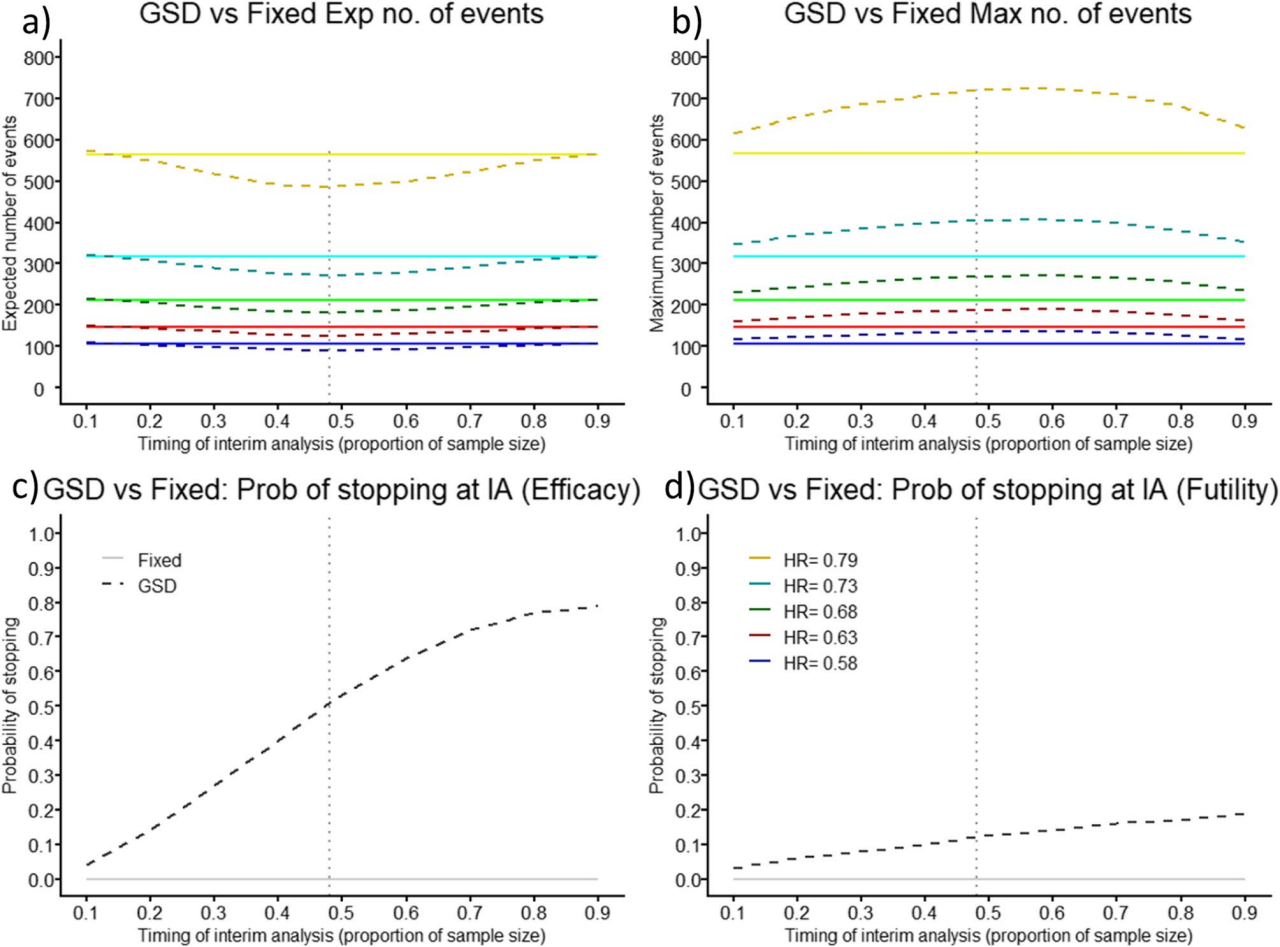
Comparison of Group Sequential Design (GSD) with Pocock boundaries (dashed) against Fixed Randomised Controlled Trial (solid) as timing of interim analysis (IA) placement changes showing (**a**) expected (Exp) number of events, (**b**) maximum (Max) number of events, (**c**) probability (Prob) of stopping for efficacy at the IA, and (**d**) Prob of stopping for futility at the IA, for five different prevention effects, for power of 80%

The optimal placement of the single IA using the Pocock boundaries is 48% of the maximum number of events, when the power is 80%, with a 51% and 12% probability of early stopping for efficacy and futility, respectively. This results in a slightly smaller expected number of events (3.4%) and patients (3.6%) compared to using the OBF boundaries, for each HR evaluated (scenario 1: Pocock = 485 vs. OBF = 502 events and Pocock = 2380 vs. OBF = 2470 patients). Conversely, the OBF boundaries produce a reduction for the maximum number of events (14.3%) and patients (10.9%) (scenario 1: Pocock = 720 vs. OBF = 617 events and Pocock = 3251 vs. OBF = 2896 patients), Fig. 2 and Table SM3 in the additional material 1. The distribution of required events for a GSD with Pocock boundaries is displayed in Figure SM4 in the additional material 1. Similar results are displayed in Figure SM5 and Table SM4 in the additional material 1, when the power is 90%. Due to the possibility of having a larger study (if the trial does not stop at the IA) when utilising the Pocock boundaries, we focus on the OBF boundaries as preferred, throughout the rest of this manuscript.

We further investigate the impact of changing the number of IAs from one to nine in a GSD utilising formal efficacy and non-binding futility OBF boundaries. Increasing the number of IA, increases the probability of early stopping for efficacy (one = 18% vs. nine = 76%) and futility (one = 7% vs. nine = 18%), which decreases the expected number of events (by one = 7.6% vs. nine = 20.1%) and expected number of patients (by one = 7.3% vs. nine = 16.7%) in comparison to the fixed RCT (scenario 1: 566 events and 2737 patients). This increases the maximum number of events (by one = 5.5% vs. nine = 19.8%) and increases the maximum number of patients (by one = 3.2% vs. nine = 13.5%) in comparison to the fixed RCT (Figures SM6-7 in the additional material 1).

### Sample size re-estimation

For SSR, we used OBF efficacy stopping boundaries placed at the optimal timing of IA from the GSD, i.e. 64% of the maximum number of events, and aimed for 80% power. Furthermore, the trial would be stopped at IA for futility if the observed HR ≥ 1. We used an assumed prevention effect 5% larger (based on a 5% larger reduction in VAP in the investigational intervention arm) than the true prevention effect used in the simulations, to calculate the number of events and patients needed in the first and second stage of the trial. Then the prevention effect observed in the simulated data at the IA and the total maximum number of events found via the optimisation function were used to recalculate the number of events and patients needed in the second stage of the trial to reach a conditional power of 80% across 10,000 simulations. See Figures SM8-SM9 in the additional material 1 for more details.

### Comparison of designs where the assumed prevention effect is correct

We investigated the SSR design for several true HRs (0.58, 0.68, 0.79), where the assumed HR used to calculate the initial sample size is purposely based on a prevention effect 5% larger than the assumed HR (HR 0.53, 0.63, 0.73, respectively) (Table 2). When we compare SSR to the GSD (with optimal IA timing, 64%) and fixed design (both using the true prevention effect), the adaptive trial designs give smaller expected numbers of events, with an increase in maximum numbers of events. For scenarios 3 and 5, the GSD produces the lowest expected number of events, while the fixed RCT produces the highest (Table 2). The adaptive designs reduce the expected number of events by 9.4%−11.3% and expected number of patients by 7.8%−9.5% in comparison to the fixed RCT. However, they increase the mean maximum number of events by 5.7%−9.4% and patients by 5.0-8.2% in comparison to the fixed RCT. For scenario 1, the increase needed in the number of events at the IA, to reach the conditional power for both SSR designs would be larger than the pre-determined limit. Therefore, if the SSR design simulations did not stop for efficacy, we stopped them for futility at the IA, producing the smallest expected and maximum number of events and patients across all designs.

**Table 2:** Comparison of Group Sequential Design (GSD), Sample Size Re-estimation (SSR)-increase only and SSR-both (utilising 10,000 simulations) with O’Brien-Fleming boundaries against Fixed Randomised Controlled Trial (RCT) with interim analysis (IA) placed at 64% of the trial, for three different prevention effects, for power of 80%. The Fixed RCT and GSD use the true hazard ratio (HR) in their sample size calculation, the SSR-increase only and SSR-both use the assumed HR for their initial sample size calculation and the HR observed in the simulated data to re-calculate the sample size at the IA

	Scenario	Assumed HR	True HR	Fixed RCT	GSD OBF	SSR -increase	SSR - both
Expected no. of events	1	0.73	0.79	566	502	206	206
Expected sample size	1	0.73	0.79	2710	2470	1226	1226
Mean Maximum no. of events	1	0.73	0.79	NA	617	206	206
Mean Maximum sample size	1	0.73	0.79	NA	2896	1226	1226
Total Maximum no. of events	1	0.73	0.79	NA	NA	206	206
Expected no. of events	3	0.63	0.68	212	188	192	190
Expected sample size	3	0.63	0.68	1291	1170	1185	1169
Mean Maximum no. of events	3	0.63	0.68	NA	231	227	224
Mean Maximum sample size	3	0.63	0.68	NA	1389	1377	1355
Total Maximum no. of events	3	0.63	0.68	NA	NA	287	296
Expected no. of events	5	0.53	0.58	106	94	96	96
Expected sample size	5	0.53	0.58	747	676	686	689
Mean Maximum no. of events	5	0.53	0.58	NA	116	113	115
Mean Maximum sample size	5	0.53	0.58	NA	807	803	808
Total Maximum no. of events	5	0.53	0.58	NA	NA	139	151

### Comparison of designs where the assumed prevention effect is incorrect

**Fig. 3:**
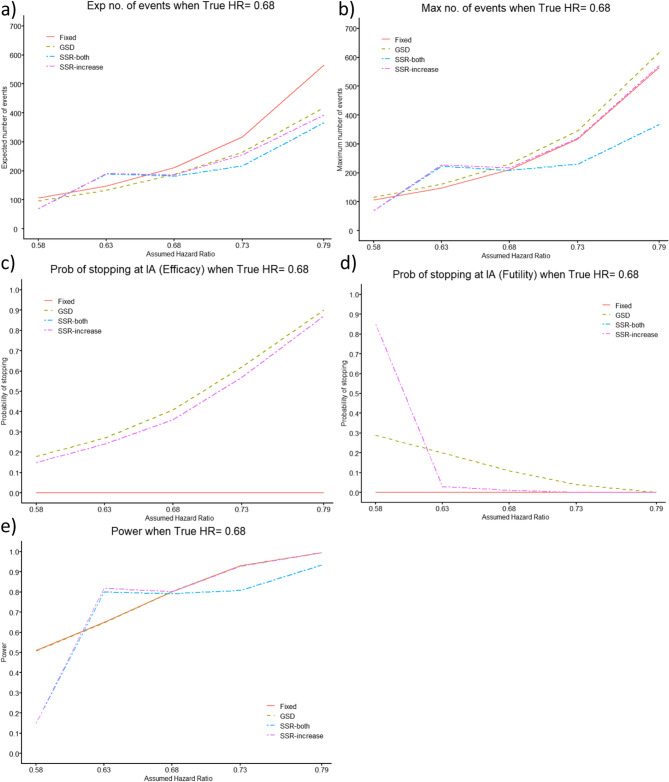
Comparison of Group Sequential Design (GSD) and Sample Size Re-estimation (SSR, utilising 5,000 simulations and an optimisation prevention effect, HR = 0.68) with O’Brien-Fleming boundaries against Fixed Randomised Controlled Trial (RCT) for (**a**) expected (Exp) number of events, (**b**) mean maximum (Max) number of events, (**c**) probability (Prob) of stopping for efficacy at the interim analysis (IA), (**d**) Prob of stopping for futility at the IA and (**e**) power, for a range of assumed HRs, when the true HR = 0.68, for power of 80%, with a single IA 64% through the trial

Figure 3 shows the situation where the assumed HR initially used for the sample size calculation (for all designs) is not equal to the true HR, and thus, the study is either underpowered, i.e. the effect of the investigational intervention was overestimated, or the effect of the SoC was underestimated or the study is overpowered, i.e. the effect of the investigational intervention was underestimated, or the effect of the SoC was overestimated.

When the assumed HR is further from the null than the true HR, the study is underpowered. The power of the GSD and fixed design is always below the target of 80%. However, the SSR designs allow the second stage sample size to increase enough, such that they attain the target power, 80%. Due to the limit we have chosen for finding the optimal total maximum number of events, the SSR designs only allow an increase in sample size when the assumed HR is relatively similar to the true HR. When the assumed HR is far from the true HR, we have a high probability of stopping the SSR designs at the IA for futility, as the increase in sample size is so large it would be more beneficial to run a completely new study, see also Figure SM10 in the additional material 1. Allowing the sample size to also decrease at the IA, requires an even larger increase in the total maximum number of events. The probability of stopping the trial early for efficacy is similar for the GSD and SSR, due to using similar efficacy stopping values. However, the probability of stopping for futility is very different, due to the different approaches used (GSD: stopping boundary vs. SSR: sample size increase being too large to be beneficial), left side of Fig. 3 and Figure SM10 in the additional material 1.

When the assumed prevention effect used to initially calculate the sample size is equal to the true prevention effect, there is little difference between the GSD and SSR, shown in Fig. 3 (assumed HR 0.68) and Figures SM 10–11 (assumed HR 0.79 and 0.58 in the additional material 1). They give an expected number of events smaller than the RCT, and a larger mean maximum number of events.

When the assumed HR utilised for the initial sample size calculation is closer to the null than the true HR, the study is overpowered. Here, the power of all trial designs, is always at least as large as the target of 80%. The SSR design allowing a decrease in sample size has a smaller increase in power than the other designs, i.e., is the most efficient approach, see also Figure SM11 in the additional material 1. The probability of stopping the trial early for efficacy and futility is similar for the GSD and both the SSR designs. The expected number of events is similar for the GSD and both SSR designs, and smaller than the fixed RCT. In addition, the GSD, SSR-increase and fixed design produce a similar mean maximum number of events, whereas SSR-both consistently produces the smallest mean maximum number of events, right side of Fig. 3 and Figure SM11 in the additional material 1.

**Fig. 4:**
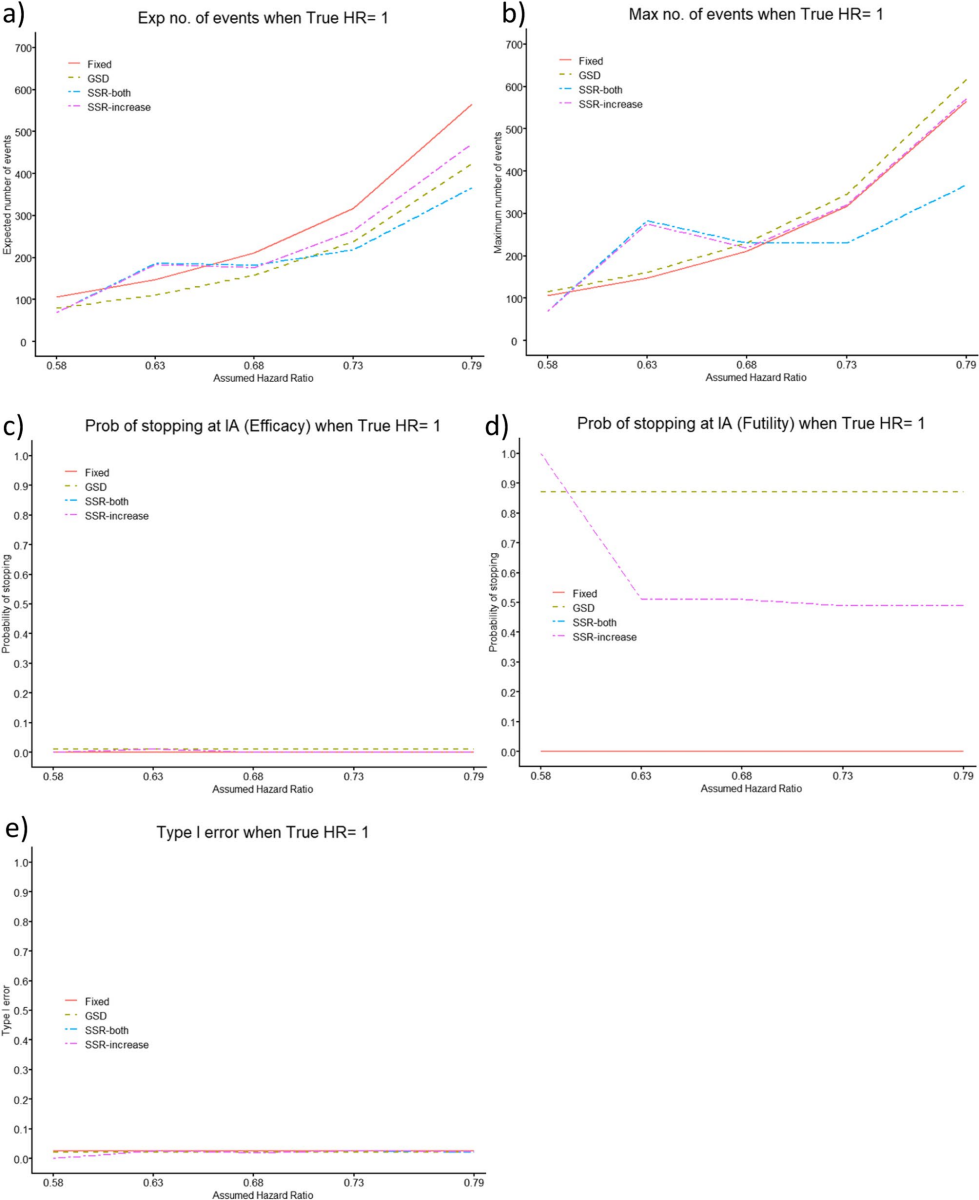
Comparison of Group Sequential Design (GSD) and Sample Size Re-estimation (SSR, utilising 5,000 simulations and an optimisation prevention effect, HR = 0.68) with O’Brien-Fleming boundaries against Fixed Randomised Controlled Trial (RCT) for (**a**) expected (Exp) number of events, (**b**) mean maximum (Max) number of events, (**c**) probability (Prob) of stopping for efficacy at the interim analysis (IA), (**d**) Prob of stopping for futility at the IA and (**e**) type I error, for a range of assumed HRs, when the true HR = 1, for desired power of 80%, with a single IA 64% of the way through the trial

Figure 4 shows the situation where the true HR is 1, and thus, the investigational intervention is no better than the SoC. To find the optimal total maximum number of events with the SSR designs, we again utilised an optimisation prevention effect of HR = 0.68. Here, the type I error (erroneously concluding the investigational intervention to be best) of all trial designs, is at most 0.025, where all four designs have a similar type I error.

The efficacy stopping probability is low for the GSD and both SSR designs and the futility stopping probability is high. The futility stopping is consistently high (87%) for the GSD across all assumed HRs, however, it decreases from 99% to roughly 50% when the assumed HR ≥ 0.63 for both SSR designs.

The GSD consistently produces a smaller expected number of events than the fixed RCT across all assumed HRs. The SSR designs give a larger expected number of events than the fixed RCT, when the assumed HR = 0.63, but a smaller expected number of events for all other assumed HRs investigated, with the SSR-both design producing the smallest. Furthermore, the SSR-both design gives a smaller expected number of events than the GSD when the assumed HR ∈ {0.58, 0.73, 0.79}.

The GSD consistently produces a slightly larger mean maximum number of events than the fixed RCT across all assumed HRs. The SSR designs give a much larger mean maximum number of events than the fixed RCT, when the assumed HR = 0.63. The SSR-increase design gives a mean maximum number of events similar to the fixed RCT for assumed HR ≥ 0.68 and smaller than the fixed RCT for assumed HR = 0.58, while the SSR-both design produces the smallest mean maximum number of events for assumed HR ∈ {0.58, 0.73, 0.79}, Fig. 4.

Figures SM12-15 in the additional material 1 demonstrate similar results when the power of the trial is targeted to be 90%.

## Discussion

In this simulation study we have explored the added value and feasibility of the GSD and SSR trial designs in comparison to a conventional fixed RCT in the setting of a trial evaluating the effectiveness of an investigational intervention vs. SoC to prevent VAP in an ICU population. Results indicate that the GSD and SSR designs are likely to decrease the expected number of events and hence, the expected sample size of a trial, reducing trial costs, when compared to the fixed RCT, while not greatly increasing the maximum number of events within the trial. To be noted, OBF boundaries gave better results than Pocock boundaries, specifically when focusing on the maximum number of events, showing the greater feasibility of utilising these boundaries. The relative gain of the adaptive designs are similar across scenarios, and for both target powers explored. SSR designs are most efficient when the true prevention effect is overestimated and hence the trial is underpowered, although, there is little difference between GSDs and SSR when the correct prevention effect is assumed. Based on these results, the application of GSD or SSR should always be considered when planning an RCT in these settings.

The general idea for the GSD is to calculate a *(relatively) large* sample size and hope to stop the trial early. Conversely, some authors suggest for SSR, we should start small and then increase the sample size appropriately at the IA [30]. Some authors recommend only allowing the sample size to increase at an IA [24]. There is much debate in the literature on which is the best approach GSD vs. SSR [10, 24, 30–32]. Here, we have shown that purposefully overestimating the prevention effect and underpowering a study in the SSR design is detrimental and utilising a GSD using the true prevention effect is more advantageous in the setting (utilising an interim analysis at 64% of the maximum number of events with OBF stopping boundaries) we have explored above (Table 2). Although, the placement of the IA within the SSR designs was not optimised, therefore, a later IA placement may improve their performance.

When little information about the prevention effect is available, SSR is advantageous, in case of under- or overestimation, however, choosing a sensible limit for the maximum increase in number of events is imperative to get maximal benefit from these designs. When the assumed prevention effect was overestimated, the GSD performed poorly. There was a small probability of correctly stopping the trial at IA for efficacy, however, often the trial would not stop and incorrectly fail to reject the null hypothesis at the final analysis, producing a low power. However, SSR could reach the desired power, by increasing the sample size of the second stage, if the assumed HR was not far from the true HR. When the assumed HR was further from the true HR, the trial could be stopped at IA for futility, saving time and patients, as the conditional power of the trial would not reach its target with our capped maximum increase in number of events. In this case, performing a new GSD would be more efficient, utilising the HR observed at IA to calculate the sample size required. In practice, sample size caps are frequently applied to avoid lengthy and costly trials and to ensure the trial is powered for a clinically meaningful effect. When the true prevention effect was underestimated and the trial was overpowered, SSR (allowing a decrease in sample size) was most efficient, as it could reach the desired power without large investments in second stage sample size. It produced the lowest mean maximum number of events and lowest expected number of events. However, it should be considered that trials need a minimum sample size to adequately assess safety outcomes and adverse events, which restricts the placement of the IA, which may limit the potential benefit of GSD and SSR.

When there was no difference between the intervention and SoC, the type I error was controlled at the alpha level for all adaptive designs, where due to the use of non-binding futility bounds, the GSD produces a conservative trial. These results represent the minimum type I error. Sometimes in clinical trial practice, although non-binding futility bounds are used, trials will not always stop early if these boundaries are crossed due to there being reasons other than efficacy, to continue the trial. In this case the type I error is inflated above what is found here, although it will still be controlled at the alpha level [23].

As we have optimised the total maximum number of events for the SSR designs considering the optimisation prevention effect, HR = 0.68, the expected number of events for the SSR-increase design are larger than the GSD when the assumed HR ≥ 0.63, this is due to the smaller probability of futility stopping for the SSR-increase design. Contrarily, the mean maximum number of events of the SSR-increase design are smaller than the GSD when the assumed HR ∈ {0.58, 0.68, 0.73, 0.79}, due to the different futility bounds used within the two trial designs. In addition, the expected and mean maximum number of events for the SSR-both design are smaller than the GSD when the assumed HR ∈ {0.58, 0.73, 0.79}, as the second stage sample size will decrease if it does not stop at the IA for futility. This shows the added benefit of allowing the SSR design to decrease the second stage sample size, when the assumed prevention effect is larger than the optimisation prevention effect.

To inform simulations for GSD or SSR to plan a specific RCT, it would be beneficial to have access to valid estimates of outcome incidence for the SoC, time from randomisation to outcome and recruitment rates from envisioned clinical study sites. This is the underlying paradigm of the perpetual observational studies (POSs), which have been designed within the European clinical research alliance on infectious diseases (ECRAID) [33]. ECRAID has set-up several POSs, which provide a permanent infrastructure of clinical sites practiced in recruiting patients and prospectively collecting high-quality data. This network would allow for efficient set-up of interventional studies, through availability of observational data to inform site selection, sample size calculations and simulations [34]. ECRAID’s POS-VAP is a network of 31 active sites (as of 18th July 2025) across twelve European countries [35], already including 6108 patients and 972 with VAP. These data and this network could help make future trials, like SAATELLITE [14] or AMIKINHAL [16], more efficient.

This study focused specifically on parallel group, two-arm, superiority trials to evaluate the effectiveness of a novel intervention to prevent VAP, inspired by SAATELLITE [14] and AMIKINHAL [16]. SAATELLITE concluded that suvratoxumab resulted in a trend towards a lower incidence of *Staphylococcus aureus* pneumonia and continued investigation of suvratoxumab in this patient population should be performed [14]. AMIKINHAL concluded that a 3-day course of inhaled amikacin reduced the burden of VAP during 28 days of follow-up, in patients who had been under IMV for at least 3 days [16]. To improve efficiency of future trials in this setting, the combination of the POS-VAP network, and informed GSD and SSR simulations should be considered.

Strengths of this work are that it was based on real-world data from HONEST-PREPS, and several different scenarios were investigated informed by results from two previous trials, SAATELITE [14] and AMIKINHAL [16]. In addition, we explored the use of these adaptive designs in the situation where our assumed prevention effect did not match the true prevention effect, a common issue for many clinical trials.

The present study also has several limitations. First, competing events have not been accounted for in the simulations. Patients at risk of developing VAP may experience a competing event such as death, ICU discharge or extubation, which means they are no longer at risk of VAP. We have minimised this limitation by utilising the cumulative incidence function to estimate the VAP rate using the HONEST-PREPS data, which did account for competing events. Furthermore, we have based this analysis on data from HONEST-PREPS, but other studies have shown differing cumulative incidences of VAP [36–38]. Thus, depending on local epidemiology and VAP definitions, VAP incidence rates and onset times may be different, which could affect the predicted benefit of these designs. Although, this further highlights the need for trials to use adaptive designs, as our prior information about the prevention effect could be incorrect.

## Conclusion

In conclusion, this simulation study shows that both GSD and SSR are effective and feasible adaptive designs for RCTs aiming to compare the effectiveness of an investigational intervention against the SoC to prevent VAP. Both designs would be preferred over a fixed RCT, as the expected number of events would be smaller, with a small probability of a slight increase in maximum number of events. Application of these adaptive designs should be considered at the trial design stage, as it would result in a more efficient RCT, reducing risks for patients within the trial, and making results available to patients faster.

## Supplementary Information

Additional file 1: Benefits of group sequential design and sample size re-estimation for RCTs evaluating the prevention of ventilator-associated pneumonia: A simulation study informed by real world data. Additional analysis results, for additional simulation scenarios.

Additional file 2: Benefits of group sequential design and sample size re-estimation for RCTs evaluating the prevention of ventilator-associated pneumonia: A simulation study informed by real world data. Document containing the R code which was used to produce the results in this manuscript.

## Data Availability

The datasets used and/or analysed during the current study will not be shared. However, the code to recreate the results of this manuscript is available in the additional material 2.

## Abbreviations

RCT: Randomised Controlled Trial
GSD: Group Sequential Design
SSR: Sample Size Re–estimation
IA: Interim Analysis
VAP: Ventilator Associated Pneumonia
IMV: Invasive Mechanical Ventilation
ICU: Intensive Care Unit
HONEST-PREPS: Hospital Network Study–Preparation for a Randomised Evaluation of anti–Pneumonia Strategies
FDA: Food and Drug Administration
Inv: Investigational intervention
SoC: Standard of Care
OBF: O’Brien Fleming
HR: Hazard Ratio
Exp: Expected
Max: Maximum
Prob: Probability
POS: Perpetual Observational Study
ECRAID: European Clinical Research Alliance on Infectious Diseases
POS-VAP: Perpetual Observational Study on Ventilator Associated Pneumonia

## References

[1] Goossens H, Derde L, Horby P, Bonten M. The European clinical research response to optimise treatment of patients with COVID-19: lessons learned, future perspective, and recommendations. Lancet Infect Dis. 2022;22:e153–8. 10.1016/S1473-3099(21)00705-2. 34951954 10.1016/S1473-3099(21)00705-2 PMC8691848

[2] Burnett T, Mozgunov P, Pallmann P, et al. Adding flexibility to clinical trial designs: an example-based guide to the practical use of adaptive designs. BMC Med. 2020;18:352. 10.1186/s12916-020-01808-2. 33208155 10.1186/s12916-020-01808-2 PMC7677786

[3] Pallmann P, Bedding AW, Choodari-Oskooei B, et al. Adaptive designs in clinical trials: why use them, and how to run and report them. BMC Med. 2018;16:29. 10.1186/s12916-018-1017-7. 29490655 10.1186/s12916-018-1017-7 PMC5830330

[4] Kairalla JA, Coffey CS, Thomann MA, et al. Adaptive trial designs: a review of barriers and opportunities. Trials. 2012;13:145. 10.1186/1745-6215-13-145. 22917111 10.1186/1745-6215-13-145 PMC3519822

[5] Van Werkhoven CH, Harbarth S, Bonten MJM. Adaptive designs in clinical trials in critically ill patients: principles, advantages and pitfalls. Intensive Care Med. 2019;45:678–82. 10.1007/s00134-018-5426-z. 30377740 10.1007/s00134-018-5426-z PMC6483961

[6] Pocock SJ. Group sequential methods in the design and analysis of clinical trials. Biometrika. 1977;64(2):191–9.

[7] Lakens D, Pahlke F, Wassmer G. Group sequential designs: A tutorial. PsyArXiv. 2021. 10.31234/osf.io/x4azm

[8] Grayling MJ, Mander AP. Accounting for variation in the required sample size in the design of group-sequential trials. Contemp Clin Trials. 2021;107:106459. 10.1016/j.cct.2021.106459. 34082076 10.1016/j.cct.2021.106459

[9] Gould AL. Sample size re-estimation: recent developments and practical considerations. Stat Med. 2001;20:2625–43. 11523073 10.1002/sim.733

[10] Pritchett YL, et al. Sample size re-estimation designs in confirmatory clinical trials—current state, statistical considerations, and practical guidance. Stat Biopharm Res. 2015;7:309–21.

[11] Chuang-Stein C, Anderson K, Gallo P, et al. Sample size reestimation: a review and recommendations. Drug Inf J. 2006;40:475–84. 10.1177/216847900604000413.

[12] Wason JMS, Brocklehurst P, Yap C. When to keep it simple – adaptive designs are not always useful. BMC Med. 2019;17:152. 10.1186/s12916-019-1391-9. 31370839 10.1186/s12916-019-1391-9 PMC6676635

[13] Chastre J, François B, Bourgeois M, et al. Safety, efficacy, and pharmacokinetics of Gremubamab (MEDI3902), an anti-*Pseudomonas aeruginosa* bispecific human monoclonal antibody, in *P. aeruginosa*-colonised, mechanically ventilated intensive care unit patients: a randomised controlled trial. Crit Care. 2022;26:355. 10.1186/s13054-022-04204-9. 36380312 10.1186/s13054-022-04204-9 PMC9666938

[14] Bruno F, et al. Efficacy and safety of suvratoxumab for prevention of Staphylococcus aureus ventilator-associated pneumonia (SAATELLITE): a multicentre, randomised, double-blind, placebo-controlled, parallel-group, phase 2 pilot trial. Lancet Infect Dis. 2021;21(9):1313–23. 33894131 10.1016/S1473-3099(20)30995-6

[15] Bruno F, et al. Safety and pharmacokinetics of an anti-PcrV pegylated monoclonal antibody fragment in mechanically ventilated patients colonized with Pseudomonas aeruginosa: a randomized, double-blind, placebo-controlled trial. Crit Care Med. 2012;40(8):2320–6. 22622405 10.1097/CCM.0b013e31825334f6

[16] Ehrmann S, Barbier F, Demiselle J, Quenot JP, Herbrecht JE, Roux D, et al. Inhaled amikacin to prevent ventilator-associated pneumonia. N Engl J Med. 2023;389(22):2052–62. 37888914 10.1056/NEJMoa2310307

[17] Dahyot-Fizelier C, Lasocki S, Kerforne T, Perrigault PF, Geeraerts T, Asehnoune K, et al. Ceftriaxone to prevent early ventilator-associated pneumonia in patients with acute brain injury: a multicentre, randomised, double-blind, placebo-controlled, assessor-masked superiority trial. Lancet Respir Med. 2024;12(5):375–85. 38262428 10.1016/S2213-2600(23)00471-X

[18] COMBACTE-NET. (February 2023) HONEST-PREPS Trial Story. Accessed 15/02/2024 at https://www.combacte.com/news/honest-preps-trial-story/

[19] Hospital Network Study – Preparation for a Randomized Evaluation of Anti-Pneumonia Strategies (HONEST-PREPS). ClinicalTrials.gov identifier: NCT05060718. Updated November 29, 2023. Accessed 07/01/2025. https://clinicaltrials.gov/study/NCT05060718

[20] Jackson H, Sauser J, Bonten M et al. (2023, September 12–15). Clinical and microbiological epidemiology of hospital-acquired and ventilator-associated pneumonia: prospective study in 12 European ICUs [Poster presentation]. International Conference on Prevention and Infection Control, Geneva, Switzerland. https://aricjournal.biomedcentral.com/articles/supplements/volume-12-supplement-1

[21] US Food and Drug Administration. (June 2020) Hospital-Acquired bacterial pneumonia and ventilator associated bacterial pneumonia: developing drugs for treatment guidance for industry. FDA, Accessed 20/02/2024, https://www.fda.gov/media/79516/download

[22] DeMets DL, Gordon Lan KK. (2014). Alpha-Spending Function. In Methods and Applications of Statistics in Clinical Trials, N. Balakrishnan, editor. 10.1002/9781118596005.ch5

[23] Li X, Herrmann C, Rauch G. Optimality criteria for futility stopping boundaries for group sequential designs with a continuous endpoint. BMC Med Res Methodol. 2020;20:274. 10.1186/s12874-020-01141-5. 33153438 10.1186/s12874-020-01141-5 PMC7643306

[24] Hsiao ST, Lingyun L, Cyrus RM. Optimal promising zone designs. Biom J. 2019;61:1175–86. 30411405 10.1002/bimj.201700308 PMC6767001

[25] Schulgen G, et al. Sample sizes for clinical trials with time-to-event endpoints and competing risks. Contemp Clin Trials. 2005;26(3):386–96. 15911472 10.1016/j.cct.2005.01.010

[26] Schoenfeld DA. Sample-size formula for the proportional-hazards regression model. Biometrics. 1983;39:499–503. 10.2307/2531021. 6354290

[27] R Core Team. (2022). R: A language and environment for statistical computing. *R Foundation for Statistical Computing, Vienna, Austria*. URL: https://www.R-project.org/. Accessed February 28, 2024.

[28] Wassmer G, Pahlke F. (2024). rpact: Confirmatory Adaptive Clinical Trial Design and Analysis. *R package version 3.5.1*, https://www.rpact.com, https://www.github.com/rpact-com/rpact, https://www.rpact-com.github.io/rpact/ ,https://www.rpact.org Accessed February 28, 2024 .

[29] Kunzmann K, Grayling MJ, Lee KM, Robertson DS, Rufibach K, Wason JMS. Conditional power and friends: the why and how of (un)planned, unblinded sample size recalculations in confirmatory trials. Stat Med. 2022;41(5):877–90. 10.1002/sim.9288. 35023184 10.1002/sim.9288 PMC9303654

[30] Jennison C, Turnbull BW. Adaptive sample size modification in clinical trials: start small then ask for more? Stat Med. 2015;34:3793–810. 10.1002/sim.6575. 26172385 10.1002/sim.6575

[31] Mehta CR, Pocock SJ. Adaptive increase in sample size when interim results are promising: a practical guide with examples. Stat Med. 2011;30:3267–84. 10.1002/sim.4102. 22105690 10.1002/sim.4102

[32] Emerson SS, Levin GP, Emerson SC. Comments on ‘Adaptive increase in sample size when interim results are promising: a practical guide with examples.’ Stat Med. 2011;30(28):3285–301. 10.1002/sim.4271. 22105691 10.1002/sim.4271

[33] European Clinical Research Alliance on Infectious Diseases. (April 2023) The warm base network. Ecraid, Accessed 28/02/2024, https://ecraid.eu/sites/default/files/2023-04/The_warm_base_network_white_paper.pdf

[34] Hassoun-Kheir N, et al. Perpetual observational studies: new strategies to support efficient implementation of observational studies and randomized trials in infectious diseases. Clin Microbiol Infect. 2022;28(12):1528–32. 10.1016/j.cmi.2022.07.024. 35940566 10.1016/j.cmi.2022.07.024 PMC9354481

[35] European Clinical Research Alliance on Infectious Diseases. POS-VAP. Ecraid, Accessed 28/10/2024, https://ecraid.eu/study/pos-vap

[36] Stoclin A, Rotolo F, Hicheri Y, et al. Ventilator-associated pneumonia and bloodstream infections in intensive care unit cancer patients: a retrospective 12-year study on 3388 prospectively monitored patients. Support Care Cancer. 2020;28:193–200. 10.1007/s00520-019-04800-6. 31001694 10.1007/s00520-019-04800-6 PMC7224052

[37] Xu Y, Lai C, Xu G, Meng W, Zhang J, Hou H, Pi H. Risk factors of ventilator-associated pneumonia in elderly patients receiving mechanical ventilation. Clin Interv Aging. 2019;14:1027–38. 10.2147/CIA.S197146. 31289438 10.2147/CIA.S197146 PMC6566835

[38] Craven DE, et al. Incidence and outcomes of ventilator-associated tracheobronchitis and pneumonia. Am J Med. 2013;126:542–9. 23561632 10.1016/j.amjmed.2012.12.012

