# Supplementary material for: Benefits of group sequential design and sample size re-estimation for randomised controlled trials evaluating the prevention of ventilator-associated pneumonia: a simulation study informed by real world data
Source: BMC Med Res Methodol. 2025 Nov 12;25:254. doi: 10.1186/s12874-025-02681-4 (PMC12613641; doi:10.1186/s12874-025-02681-4)
Supplement: Supplementary file 1 — Additional file 1: Benefits of group sequential design and sample size re-estimation for RCTs evaluating the prevention of ventilator-associated pneumonia: A simulation study informed by real world data. Additional analysis results, for additional simulation scenarios. [file 12874_2025_2681_MOESM1_ESM.docx]

**Additional Material 1 for *Benefits of group sequential design and sample size re-estimation for RCTs evaluating the prevention of ventilator-associated pneumonia: A simulation study informed by real world data***

Please find here additional analyses comparing the group sequential design and sample size re-estimation with the conventional fixed randomised controlled trial. These analyses are additional to what is included in the main text.

**Group Sequential Designs**

b)

c)

a)

**
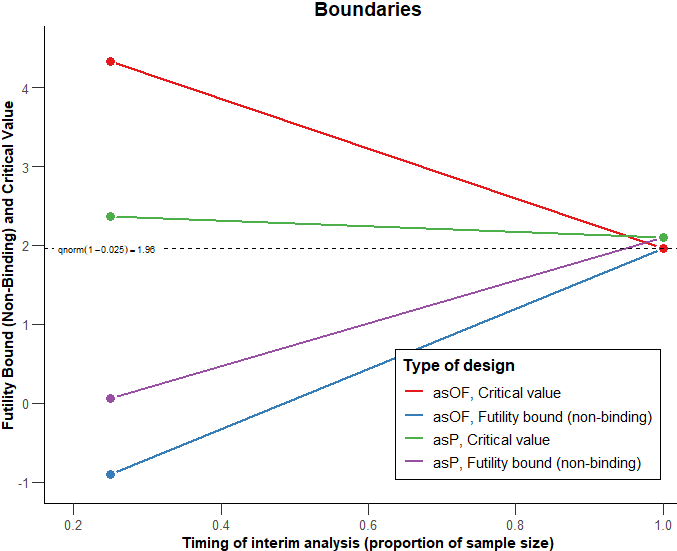
** **
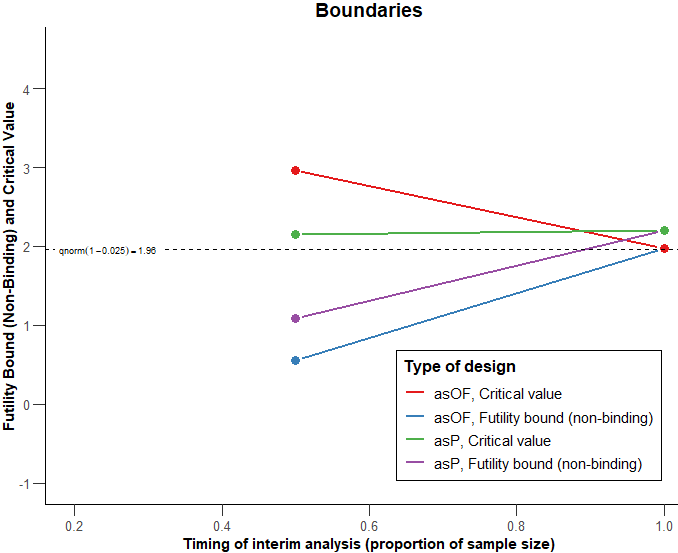
**
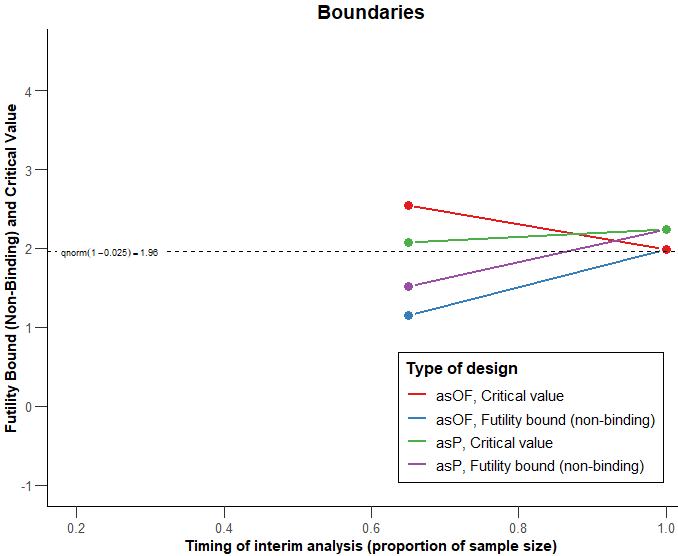


*Figure SM1: Comparison of Pocock (P) and O’Brien-Fleming (OF) efficacy and non-binding futility alpha spending (as) functions used within a Group Sequential Design with 1 interim analysis placed at (a) 25%, (b) 50% and (c) 65% of the total trial, for one-sided type I error of 2.5% and power of 80%.*

| **Scenario** | **HR** | **Fixed RCT** | | **GSD: OBF Boundaries** | | | | | | |
| --- | --- | --- | --- | --- | --- | --- | --- | --- | --- | --- |
|  |  | **no. of events** | **sample size** | **optimal IA** | **probability of stopping efficacy** | **probability of stopping futility** | **exp no. of events** | **exp sample size** | **max no. of events** | **max sample size** |
| **1** | 0.79 | 566 | 2710 | 0.64 | 41% | 11% | 502 | 2470 | 617 | 2896 |
| **2** | 0.73 | 317 | 1764 | 0.64 | 41% | 11% | 282 | 1602 | 346 | 1893 |
| **3** | 0.68 | 212 | 1291 | 0.64 | 41% | 11% | 188 | 1170 | 231 | 1389 |
| **4** | 0.63 | 148 | 972 | 0.64 | 41% | 11% | 131 | 880 | 161 | 1048 |
| **5** | 0.58 | 106 | 747 | 0.64 | 41% | 11% | 94 | 676 | 116 | 807 |

*Table SM1: Comparison of Group Sequential Design with O’Brien-Fleming boundaries against Fixed Randomised Controlled Trial at optimal interim analysis placement, for five different treatment effects, for power of 80%.*

*
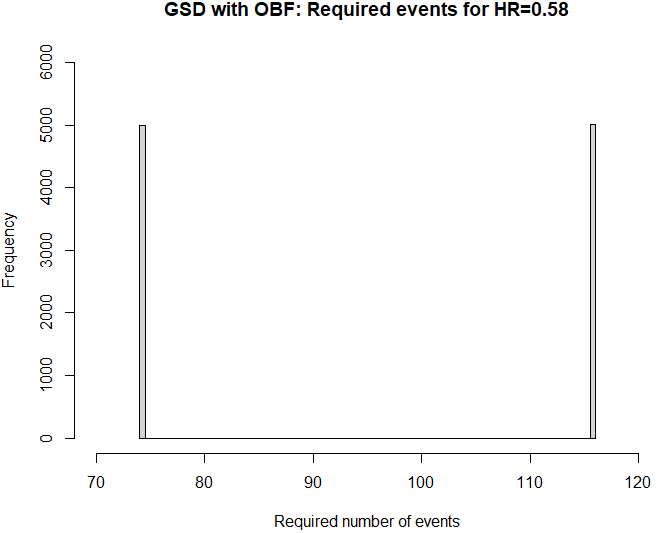
* *
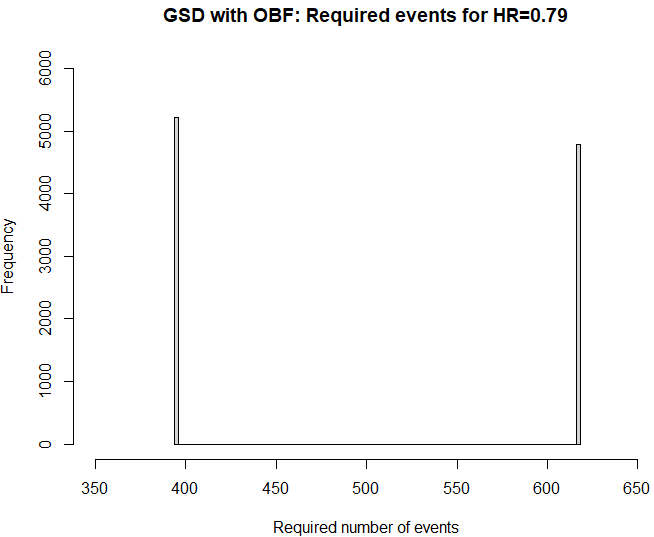
*

*Figure SM2: Frequency of total number of observed events for 10,000 simulations of a Group Sequential Design with O’Brien-Fleming boundaries with (a) HR of 0.58 and (b) HR of 0.79, with a single interim analysis placed at 64% of the way through the trial, and a power of 80%.*

**
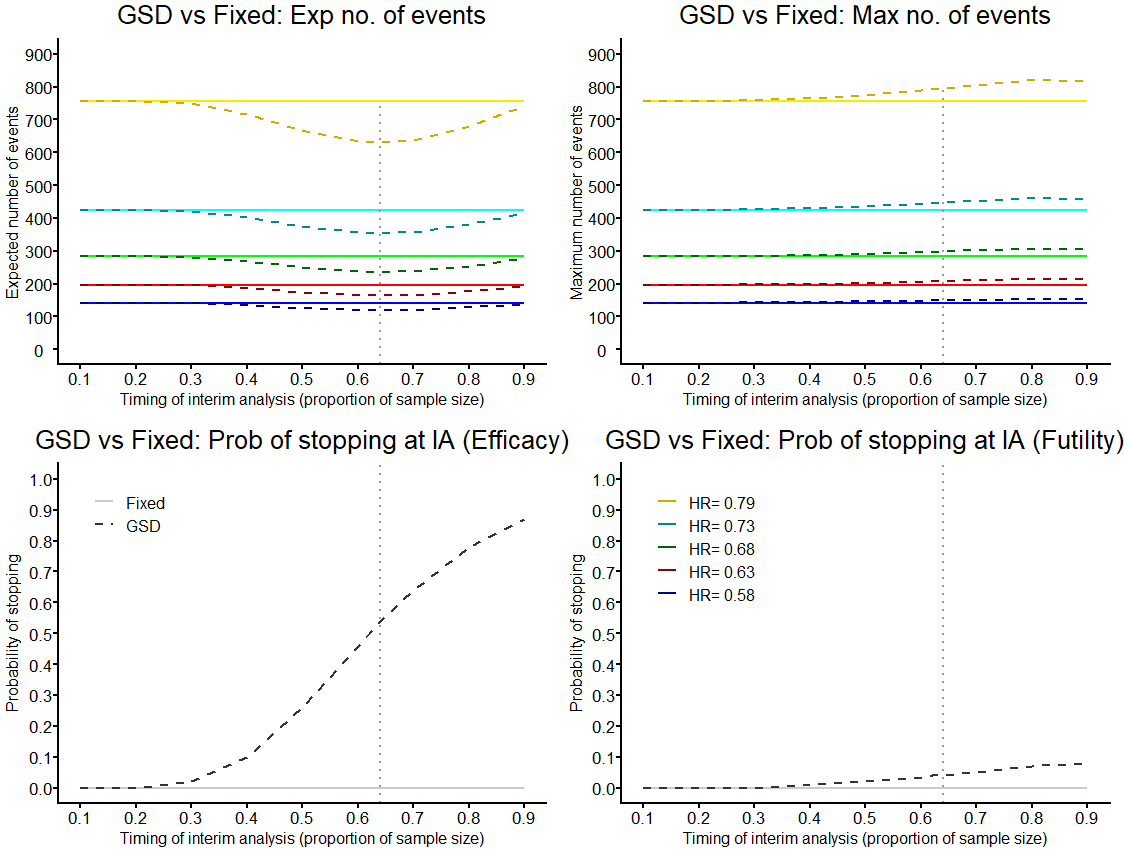
***Figure SM3: Comparison of Group Sequential Design with O’Brien-Fleming boundaries (dashed) against Fixed Randomised Controlled Trial (solid) as interim analysis (IA) placement changes for (a) expected number of events, (b) maximum number of events, (c) probability (Prob) of stopping for efficacy at the IA and (d) Prob of stopping for futility at the IA, for five different prevention effects, for power of 90%.*

d)

c)

a)

b)

| **Scenario** | **HR** | **Fixed RCT** | | **GSD: OBF Boundaries** | | | | | | |
| --- | --- | --- | --- | --- | --- | --- | --- | --- | --- | --- |
|  |  | **no. of events** | **sample size** | **optimal IA** | **probability of stopping efficacy** | **probability of stopping futility** | **exp no. of events** | **exp sample size** | **max no. of events** | **max sample size** |
| **1** | 0.79 | 757 | 3403 | 0.64 | 54% | 4% | 631 | 2942 | 796 | 3503 |
| **2** | 0.73 | 425 | 2250 | 0.64 | 54% | 4% | 354 | 1929 | 447 | 2315 |
| **3** | 0.68 | 283 | 1666 | 0.64 | 54% | 4% | 236 | 1419 | 298 | 1713 |
| **4** | 0.63 | 197 | 1267 | 0.64 | 54% | 4% | 165 | 1073 | 208 | 1302 |
| **5** | 0.58 | 142 | 983 | 0.64 | 54% | 4% | 119 | 829 | 150 | 1009 |

*Table SM2: Comparison of Group Sequential Design with O’Brien-Fleming boundaries against Fixed Randomised Controlled Trial at optimal interim analysis placement, for five different treatment effects, for power of 90%.*

| **Scenario** | **HR** | **Fixed RCT** | | **GSD: Pocock Boundaries** | | | | | | |
| --- | --- | --- | --- | --- | --- | --- | --- | --- | --- | --- |
|  |  | **no. of events** | **sample size** | **optimal IA** | **probability of stopping efficacy** | **probability of stopping futility** | **exp no. of events** | **exp sample size** | **max no. of events** | **max sample size** |
| **1** | 0.79 | 566 | 2737 | 0.48 | 51% | 12% | 485 | 2380 | 720 | 3251 |
| **2** | 0.73 | 317 | 1787 | 0.48 | 51% | 12% | 272 | 1544 | 404 | 2139 |
| **3** | 0.68 | 212 | 1311 | 0.48 | 51% | 12% | 181 | 1128 | 269 | 1578 |
| **4** | 0.63 | 148 | 989 | 0.48 | 51% | 12% | 127 | 848 | 188 | 1195 |
| **5** | 0.58 | 106 | 763 | 0.48 | 51% | 12% | 91 | 652 | 135 | 924 |

*Table SM3: Comparison of Group Sequential Design with Pocock boundaries against Fixed Randomised Controlled Trial at optimal interim analysis placement, for five different treatment effects, for power of 80%.*

*
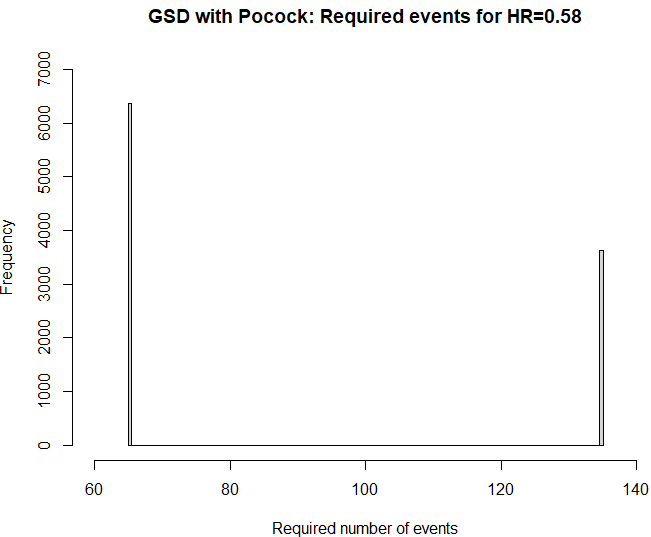
* *
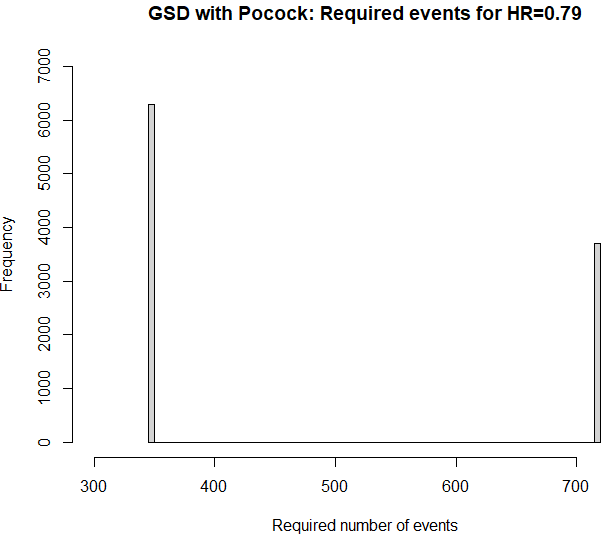
*

*Figure SM4: Frequency of total number of observed events for 10,000 simulations of a Group Sequential Design with Pocock boundaries with the “true” (a) HR of 0.58 and (b) HR of 0.79, with a single interim analysis placed at 64% of the way through the trial, and a power of 80%.*

**
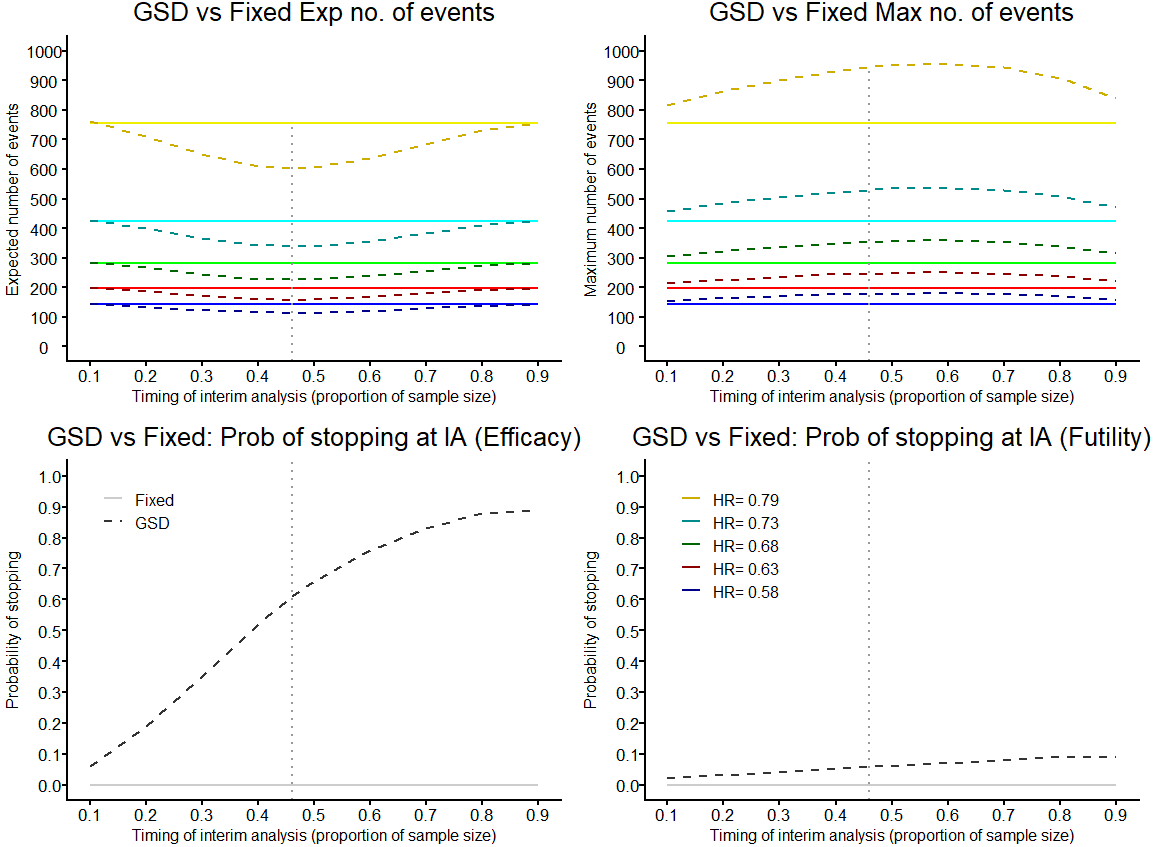
**

d)

c)

b)

a)

*Figure SM5:* *Comparison of Group Sequential Design with Pocock boundaries (dashed) against Fixed Randomised Controlled Trial (solid) as interim analysis (IA) placement changes for (a) expected number of events, (b) maximum number of events, (c) probability (Prob) of stopping for efficacy at the IA and (d) Prob of stopping for futility at the IA, for five different treatment effects, for power of 90%.*

| **Scenario** | **HR** | **Fixed RCT** | | **GSD: Pocock boundaries** | | | | | | |
| --- | --- | --- | --- | --- | --- | --- | --- | --- | --- | --- |
|  |  | **no. of events** | **sample size** | **optimal IA** | **probability of stopping efficacy** | **probability of stopping futility** | **exp no. of events** | **exp sample size** | **max no. of events** | **max sample size** |
| **1** | 0.79 | 757 | 3403 | 0.46 | 61% | 6% | 605 | 2814 | 945 | 3969 |
| **2** | 0.73 | 425 | 2250 | 0.46 | 61% | 6% | 340 | 1843 | 530 | 2641 |
| **3** | 0.68 | 283 | 1666 | 0.46 | 61% | 6% | 226 | 1355 | 353 | 1965 |
| **4** | 0.63 | 197 | 1267 | 0.46 | 61% | 6% | 158 | 1025 | 246 | 1500 |
| **5** | 0.58 | 142 | 983 | 0.46 | 61% | 6% | 114 | 792 | 177 | 1168 |

*Table SM4: Comparison of Group Sequential Design with Pocock boundaries against Fixed Randomised Controlled Trial at optimal interim analysis placement, for five different treatment effects, for power of 90%.*

**
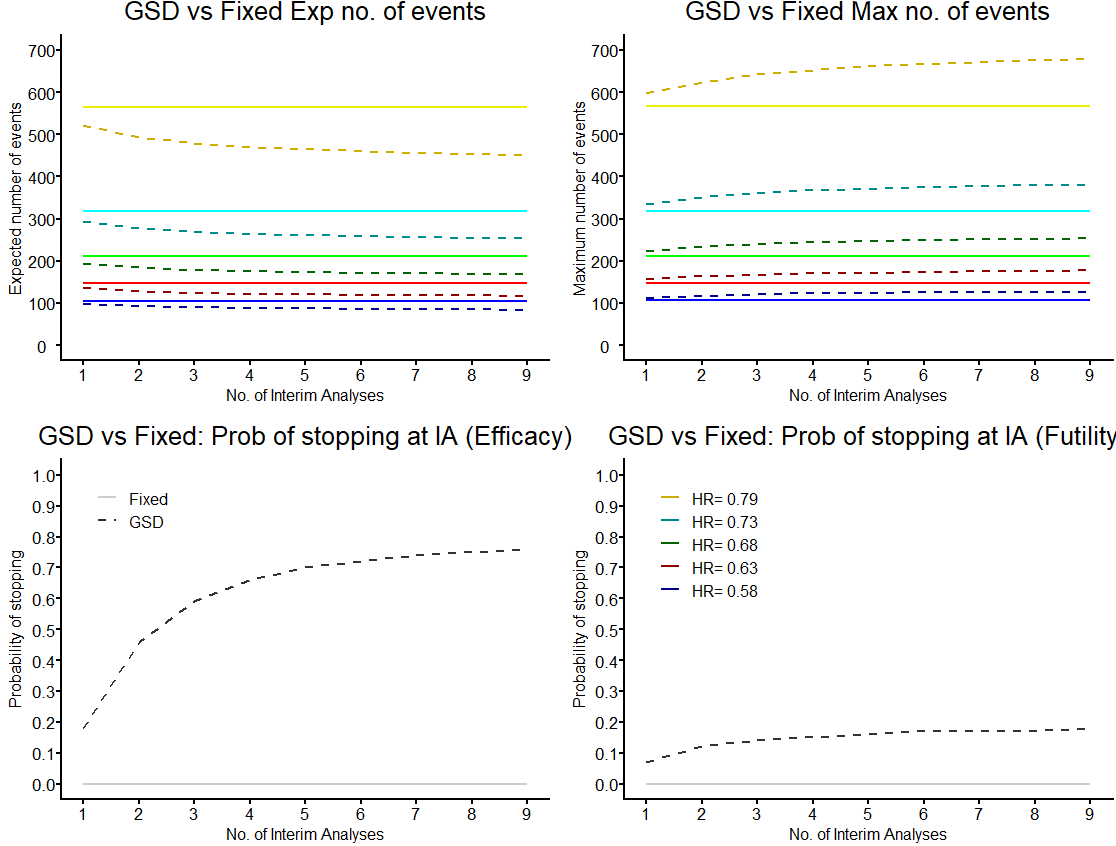
**

d)

c)

b)

a)

*Figure SM6: Comparison of Group Sequential Design with O’Brien-Fleming boundaries (dashed) against Fixed Randomised Controlled Trial (solid) as no. of interim analyses changes for (a) expected number of events, (b) maximum number of events, (c) probability (Prob) of stopping for efficacy at any IA and (d) Prob of stopping for futility at any IA, for five different treatment effects, for power of 80%.*

*
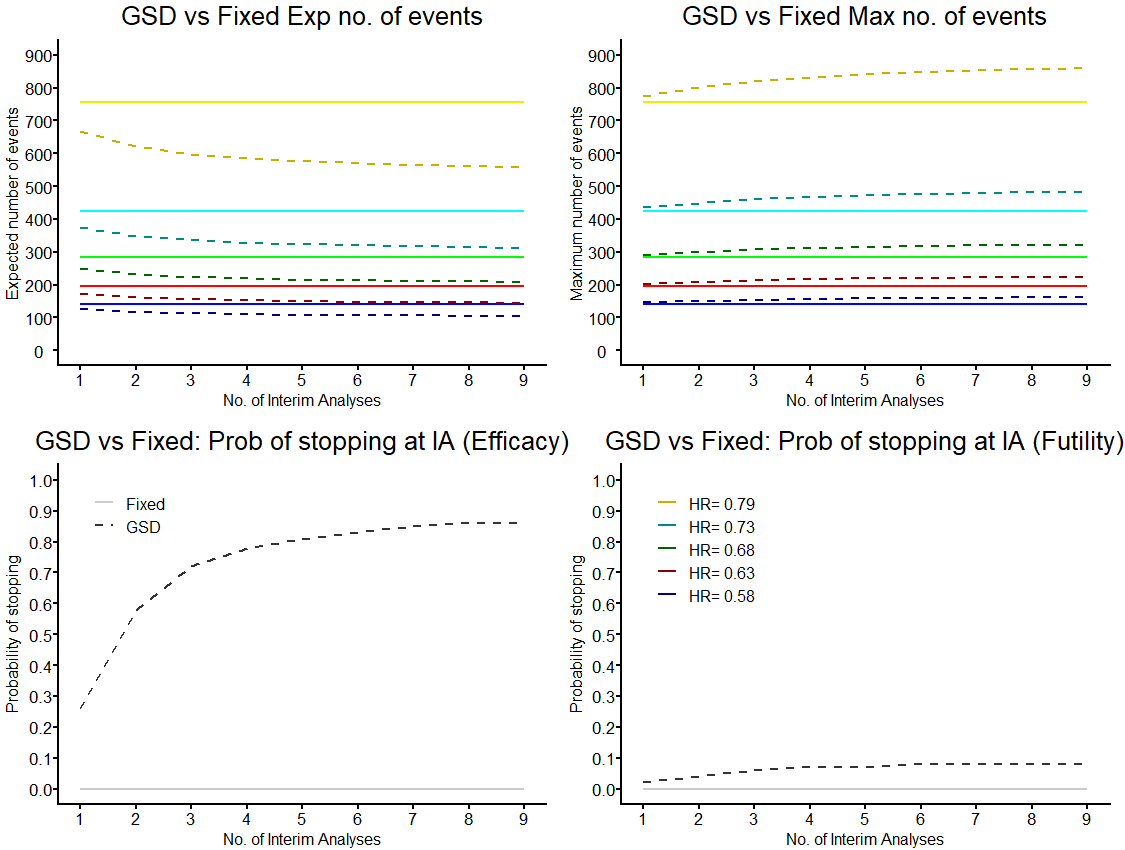
*

d)

c)

b)

a)

*Figure SM7: Comparison of Group Sequential Design with O’Brien-Fleming boundaries (dashed) against Fixed Randomised Controlled Trial (solid) as no. of interim analyses changes for (a) expected number of events, (b) maximum number of events, (c) probability (Prob) of stopping for efficacy at any IA and (d) Prob of stopping for futility at any IA, for five different treatment effects, for power of 90%.*

**Sample size Re-estimation**

As an example, a HR of 0.63 requires the single IA to be planned after observing 96 events (676 patients) and a final analysis at a total of 149 events (981 patients). The HR observed (random) at the IA will be used to recalculate the second stage sample size, this is based on a true HR of 0.68 (scenario 3), i.e. a smaller prevention effect than originally utilised or an underpowered study. The SSR-increase only design needs a total maximum number of observed events of 191 in the second stage, to reach a conditional power of 80%, resulting in a *total* maximum number of events of 287 (Figure 3) (smaller than our limit of 2*148=296). This total maximum number of events is found via an optimisation function. We use 2,000 simulations and a clinically relevant prevention effect equal to the true prevention effect, HR=0.68, to find the total maximum number of events which would allow 80% of simulations to correctly reject the null hypothesis at either stage. We further allow the trial to stop at IA for futility, if the observed HR ≥ 1. The SSR-increase design is performed over 10,000 simulations, utilising this optimised total maximum number of events. Averaging the maximum number of events across the 7,308 simulations which do not stop at the IA (for efficacy or futility) produces a *mean* maximum number of events of 227.


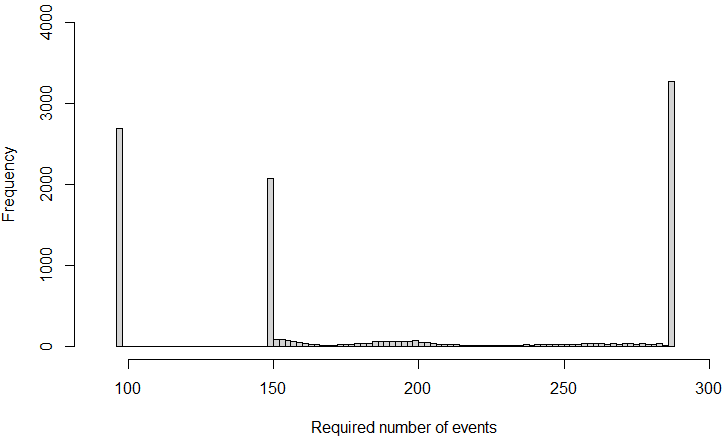


*Figure SM8: Frequency of total number of observed events for 10,000 simulations of a sample size re-estimation (increase only) trial with O’Brien-Fleming boundaries, initial assumed HR of 0.63 and the “true” optimisation prevention effect, HR of 0.68, with a single interim analysis placed at 64% of the way through the trial, and a power of 80%.*

- 2692/10,000 times (26.9%), the study stopped at the IA with 96 events
  - 2392 times (88.9%), it stopped for efficacy
  - 300 times (11.1%), null hypothesis was not rejected
- 2006/10,000 times (20.1%), the study did not increase the second stage sample size (final analysis after 149 total events)
- 1754 times (87.4%), it stopped for efficacy
- 252 times (12.6%), null hypothesis was not rejected
- 2031/10,000 times (20.3%), second stage increased between 1 and 132 events.
- 1722 times (84.8%) it stopped for efficacy
- 309 times (15.2%) significance not reached
- Remaining 3271/10,000 times (32.7%), the study reached the maximal, total no. of events, i.e. 287.
- 2299 times (70.3%) it stopped for efficacy
- 972 times (29.7%) significance not reached
- Conditional power: (2392+1754+1722+2299)/10,000=81.7%

The SSR-both design needs a total maximum number of observed events of 200 in the second stage, to reach a conditional power of 80%, resulting in a *total* maximum number of events of 296 (Figure 3) (equal to our limit of 2*148=296). This was found via an optimisation function, utilising 2,000 simulations and a clinically relevant prevention effect equal to the true prevention effect, HR=0.68. The SSR-both design is performed over 10,000 simulations, utilising this optimised total maximum number of events. Averaging the maximum number of events across the 7,308 simulations which do not stop at the IA (for efficacy or futility) produces a *mean* maximum number of events of 224.


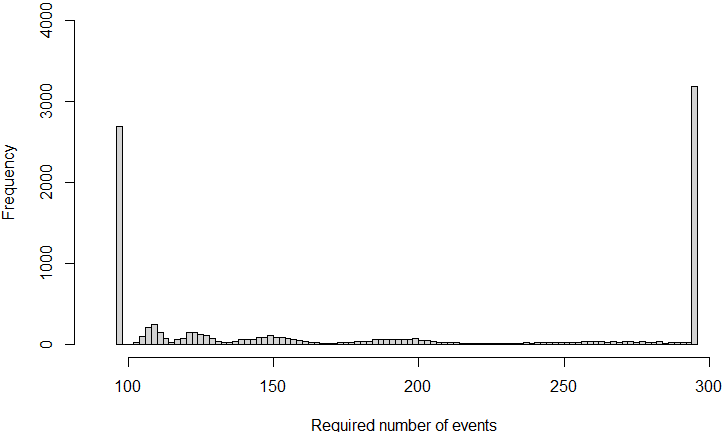


*Figure SM9: Frequency of total number of observed events for 10,000 simulations of a sample size re-estimation (both) trial with O’Brien-Fleming boundaries, initial assumed HR of 0.63 and the “true” optimisation prevention effect, HR of 0.68, with a single interim analysis placed at 64% of the way through the trial, and a power of 80%.*

- 2692/10,000 times (26.9%), the study stopped at the IA with 96 events.
  - 2392 times (88.9%), it stopped for efficacy
  - 300 times (11.1%), null hypothesis was not rejected
- 1957/10,000 times (19.6%), second stage sample size decreased.
- 1472 times (75.2%) it stopped for efficacy
- 485 times (24.8%) significance not reached
- 49/10,000 times (0.5%), the study did not increase the second stage sample size (final analysis after 149 total events).
- 44 times (89.8%), it stopped for efficacy
- 5 times (10.2%), null hypothesis was not rejected
- 2122/10,000 times (21.2%), second stage increased between 1 and 147 events.
- 1805 times (85.1%) it stopped for efficacy
- 317 times (14.9%) significance not reached
- Remaining 3180/10,000 times (31.8%), the study reached the maximal, total no. of events, i.e. 296.
- 2274 times (71.5%) it stopped for efficacy
- 906 times (28.5%) significance not reached
- Conditional power: (2392+1472+44+1805+2274)/10,000=79.9%

**Comparison of designs where the assumed prevention effect is incorrect**


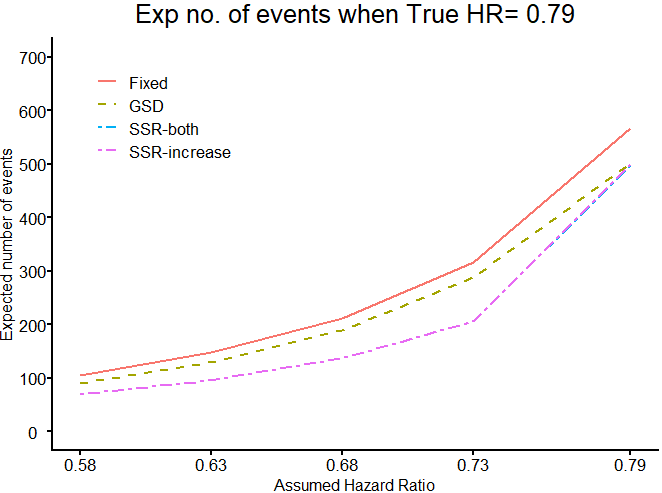

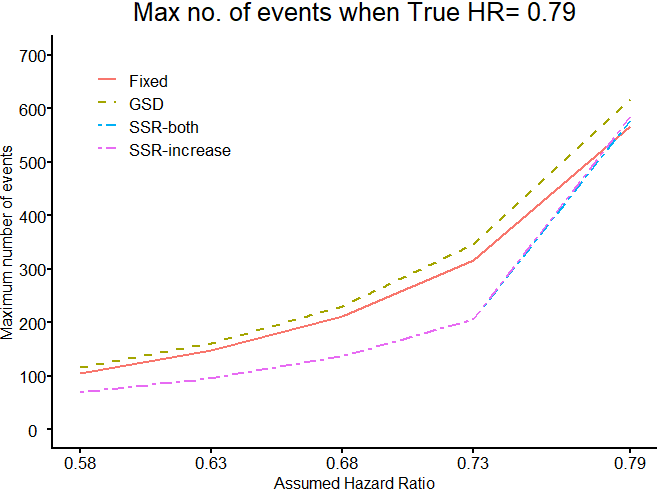

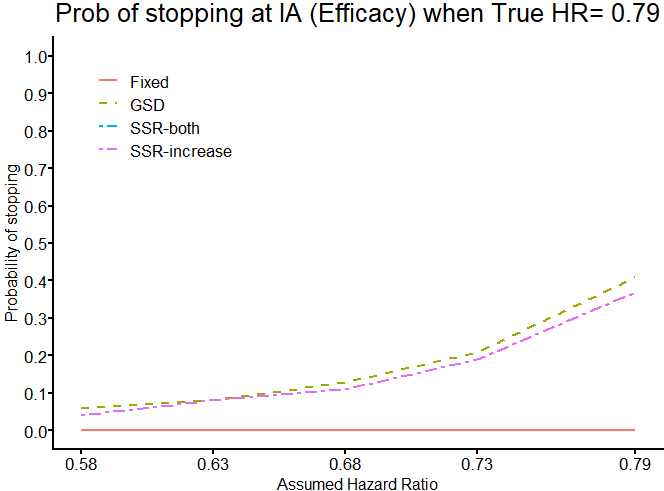

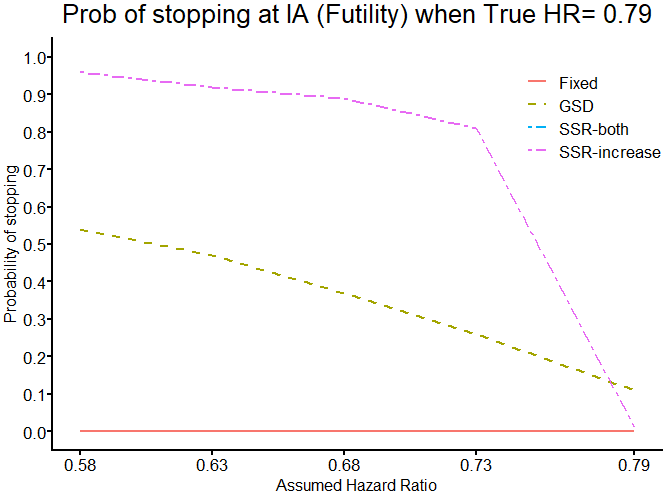

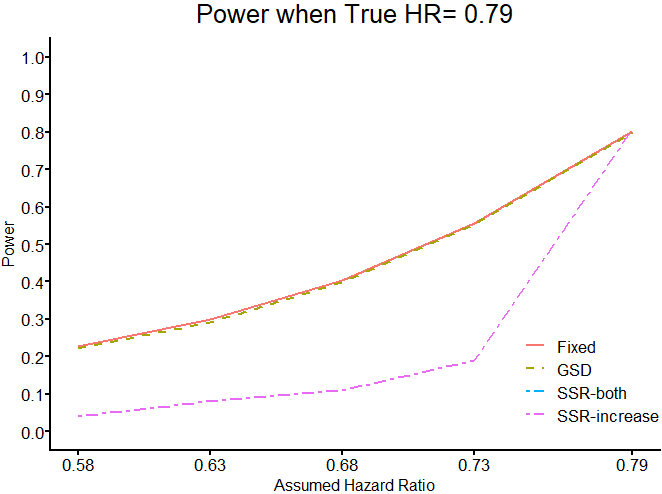


d)

e)

c)

b)

a)

*Figure SM10: Comparison of Group Sequential Design (GSD) and Sample Size Re-estimation (SSR, utilising 5,000 simulations* *and an optimisation prevention effect, HR=0.79) with O’Brien-Fleming boundaries against Fixed Randomised Controlled Trial for (a) expected number of events, (b) maximum number of events, (c) probability (Prob) of stopping for efficacy at the interim analysis (IA), (d) Prob of stopping for futility at the IA and (e) power, for five different prior HRs when the true HR=0.79, for power of 80%, with a single IA placed at 64% of the way through the trial.*


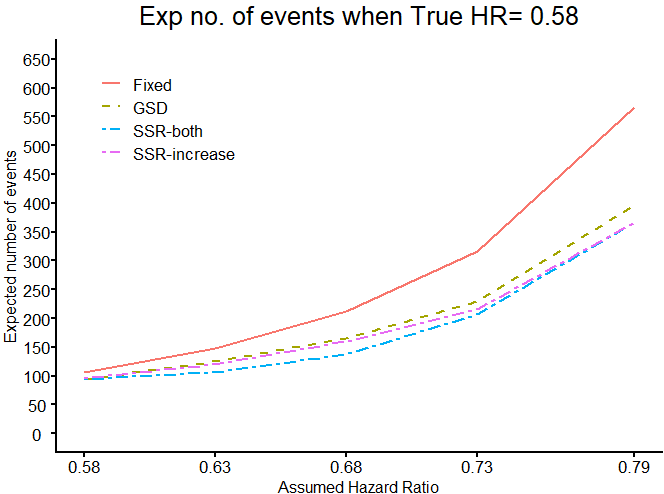

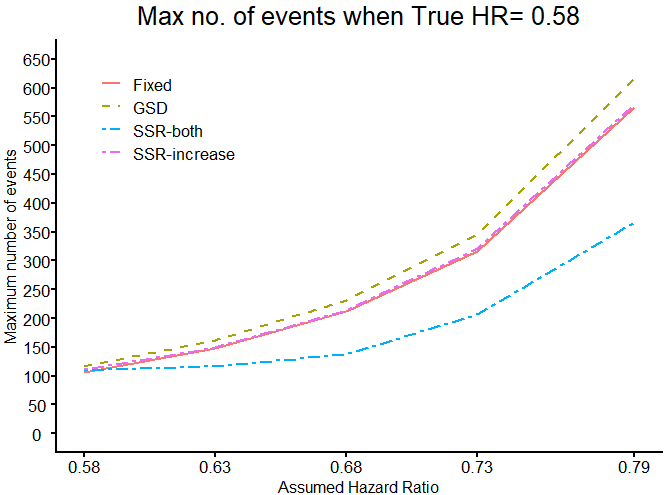

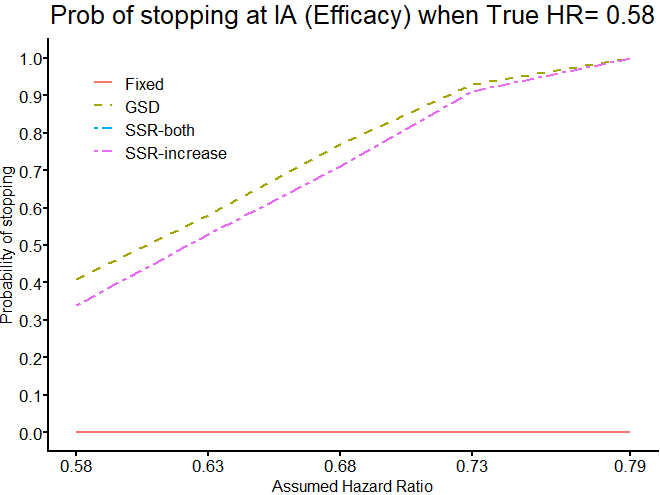

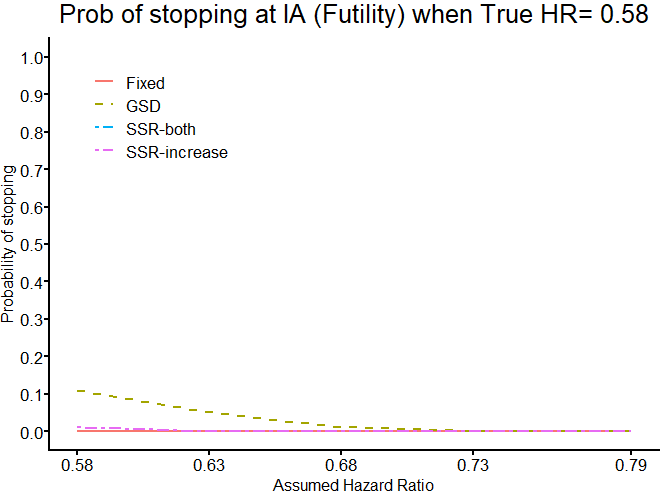

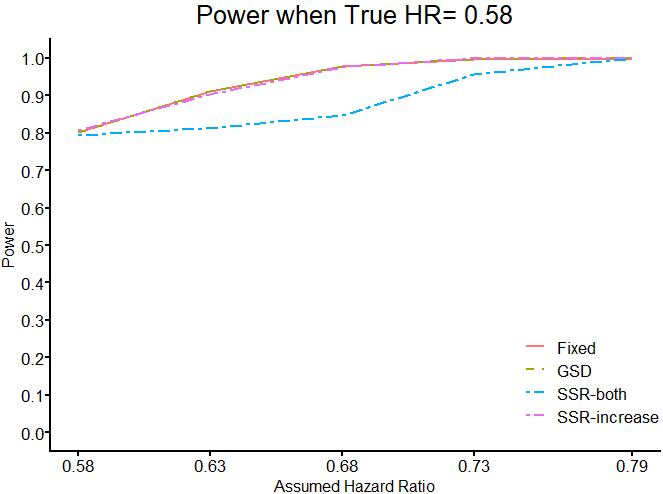


d)

e)

c)

b)

a)

*Figure SM11: Comparison of Group Sequential Design (GSD) and Sample Size Re-estimation (SSR, utilising 5,000 simulations and an optimisation prevention effect, HR=0.58) with O’Brien-Fleming boundaries against Fixed Randomised Controlled Trial for (a) expected number of events, (b) maximum number of events, (c) probability (Prob) of stopping for efficacy at the interim analysis (IA), (d) Prob of stopping for futility at the IA and (e) power, for five different prior HRs when the true HR=0.58, for power of 80%, with a single IA placed at 64% of the way through the trial.*

*
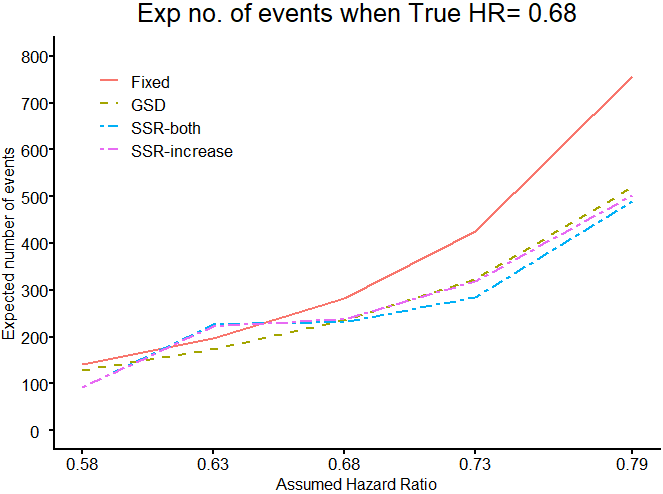

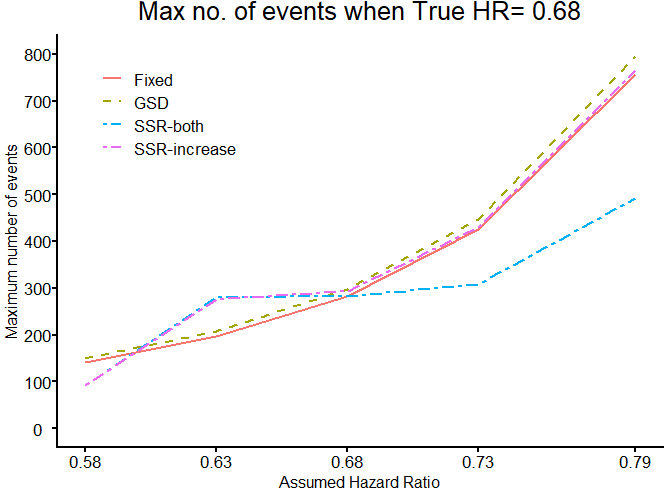

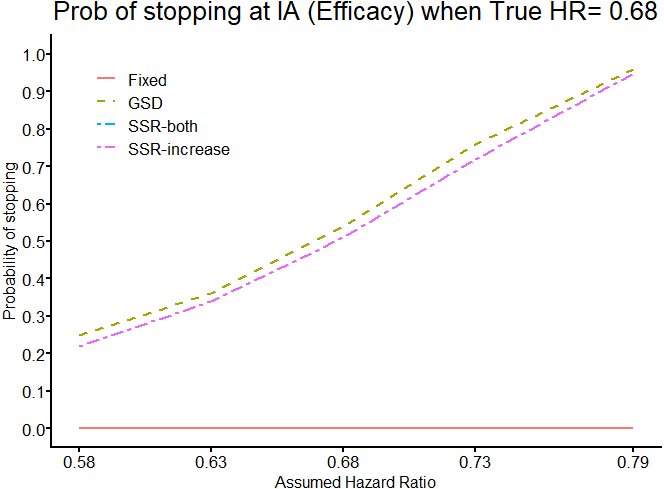

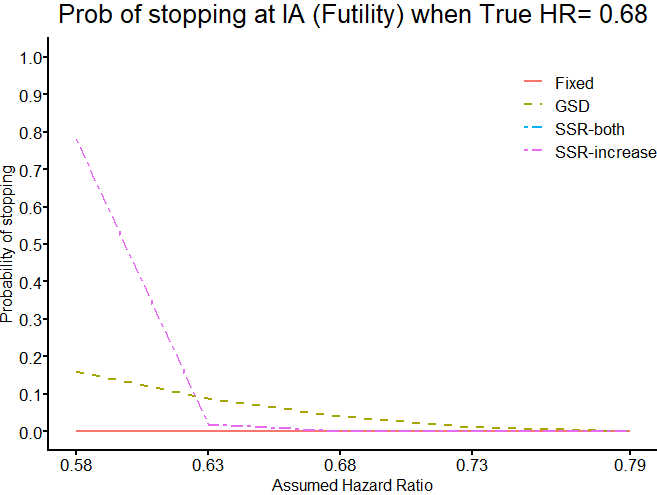

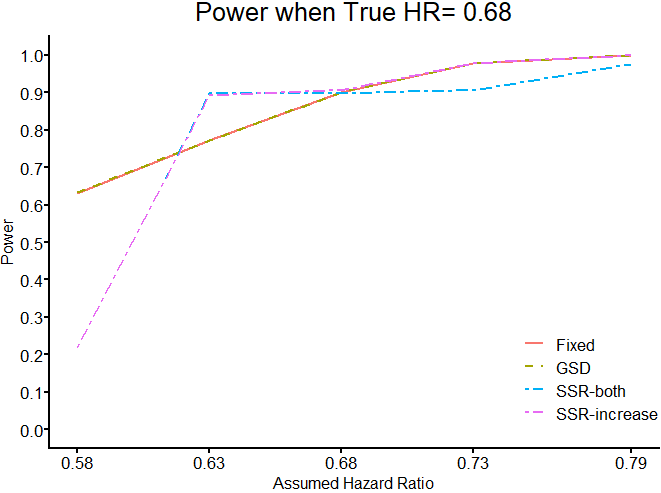
*

b)

a)

e)

c)

d)

*Figure SM12: Comparison of Group Sequential Design (GSD) and Sample Size Re-estimation (SSR, utilising 5,000 simulations and an optimisation prevention effect, HR=0.68) with O’Brien-Fleming boundaries against Fixed Randomised Controlled Trial for (a) expected number of events, (b) maximum number of events, (c) probability (Prob) of stopping for efficacy at the interim analysis (IA), (d) Prob of stopping for futility at the IA and (e) power, for five different prior HRs when the true HR=0.68, for power of 90%, with a single IA placed at 64% of the way through the trial.*


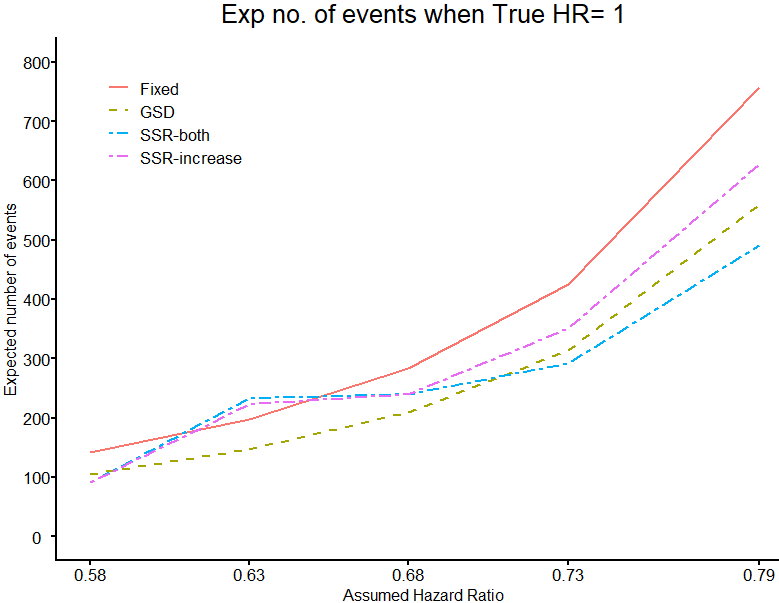

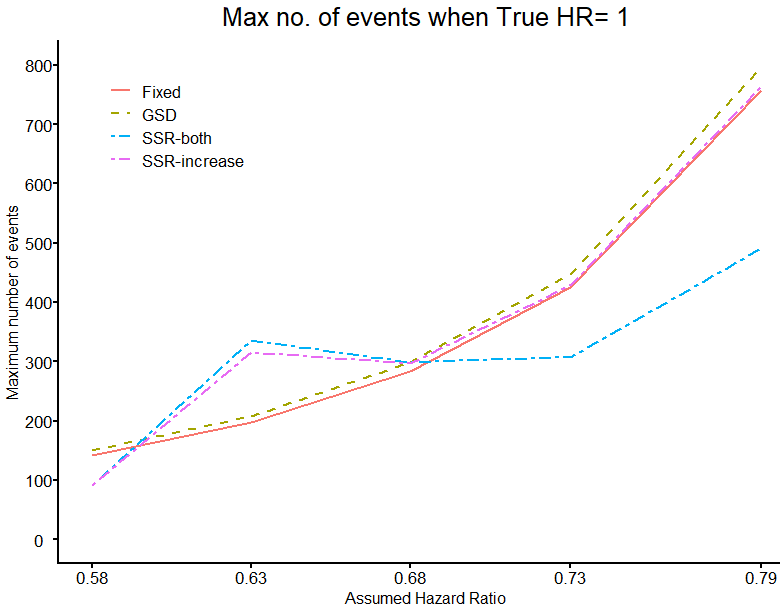

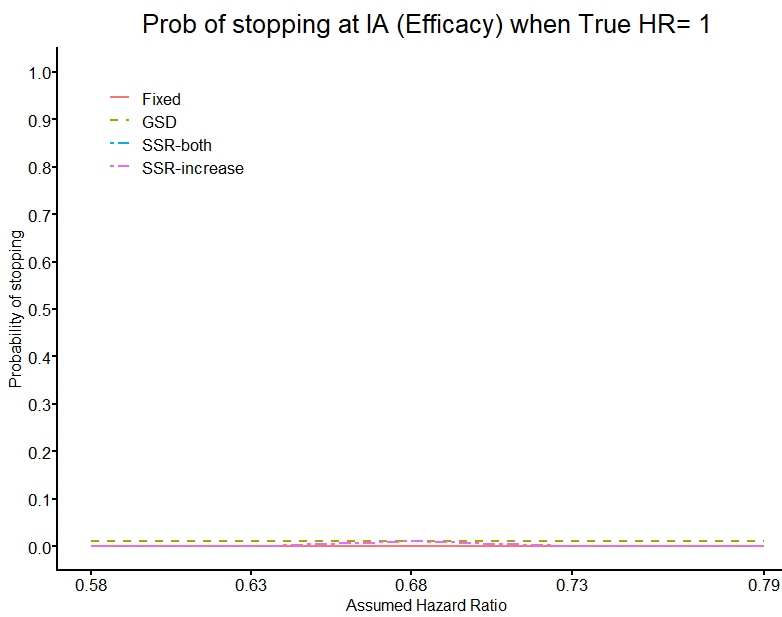

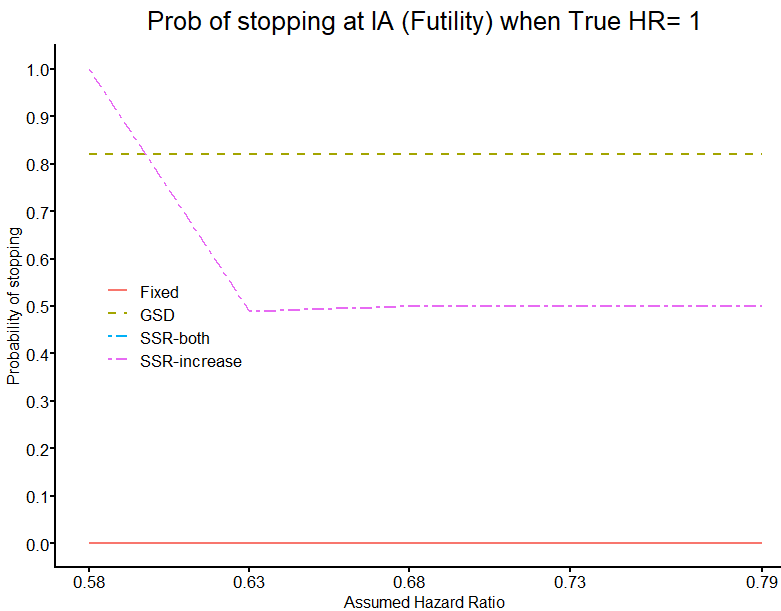

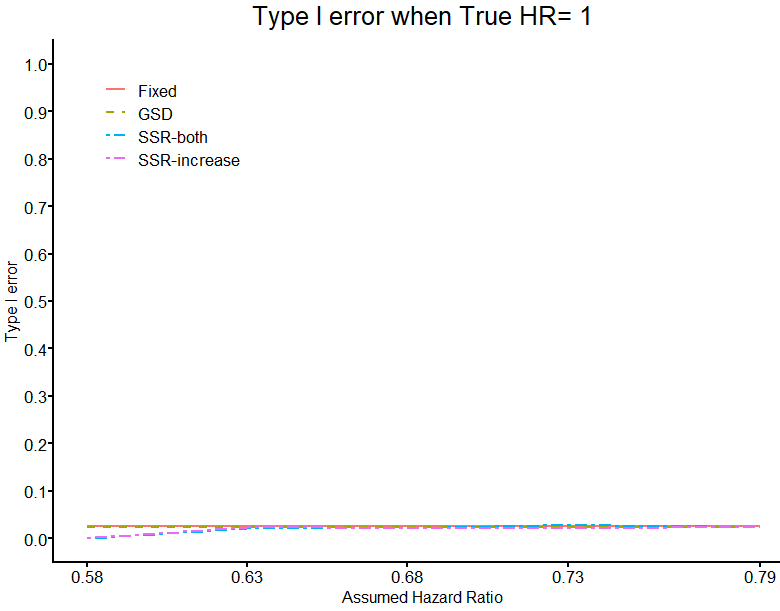


e)

c)

a)

b)

d)

*Figure SM13: Comparison of Group Sequential Design (GSD) and Sample Size Re-estimation (SSR, utilising 5,000 simulations and an optimisation prevention effect, HR=0.68) with O’Brien-Fleming boundaries against Fixed Randomised Controlled Trial for (a) expected number of events, (b) maximum number of events, (c) probability (Prob) of stopping for efficacy at the interim analysis (IA), (d) Prob of stopping for futility at the IA and (e) type I error, for five different prior HRs when the true HR=1, for desired power of 90%, with a single IA placed at 64% of the way through the trial.*

*
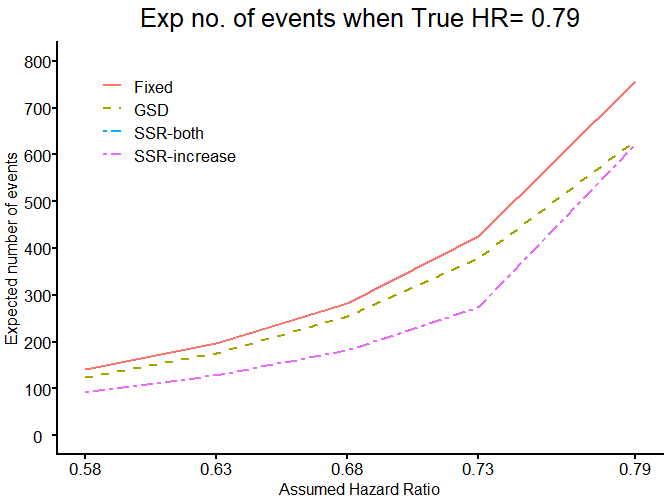

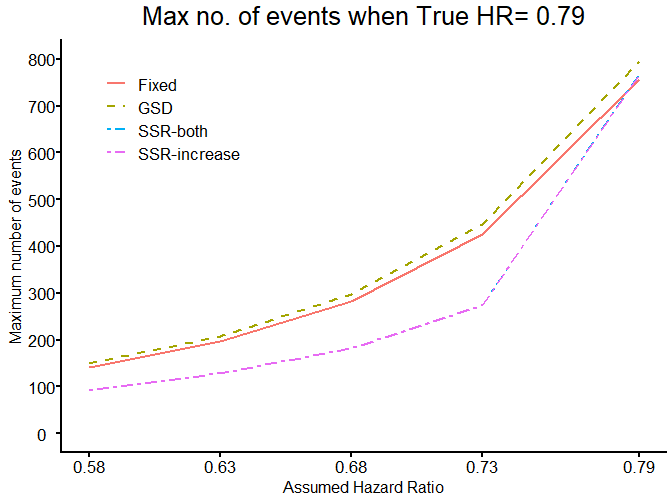

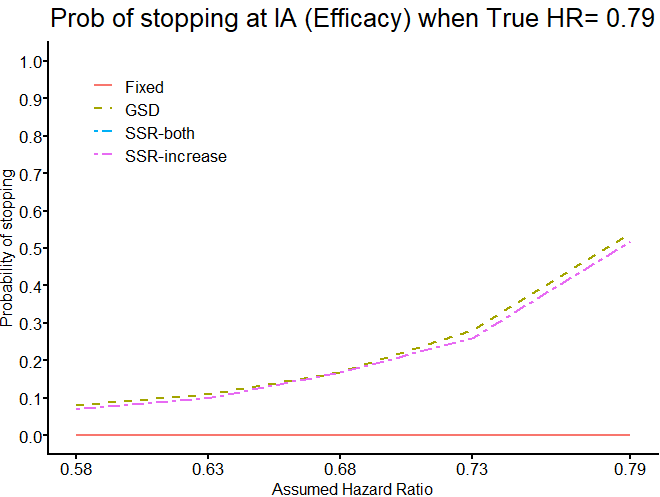

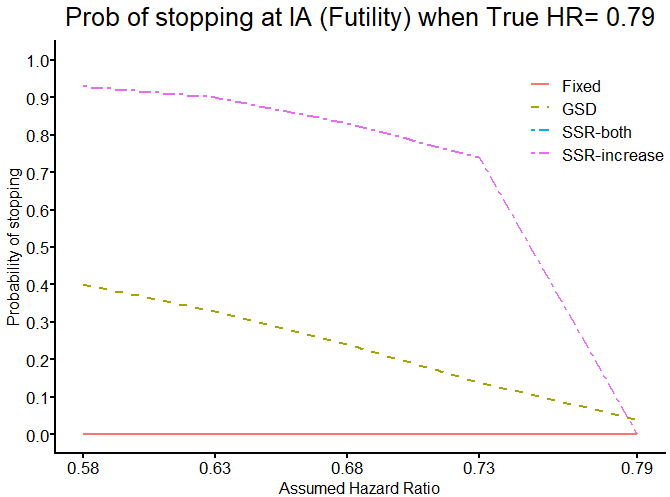

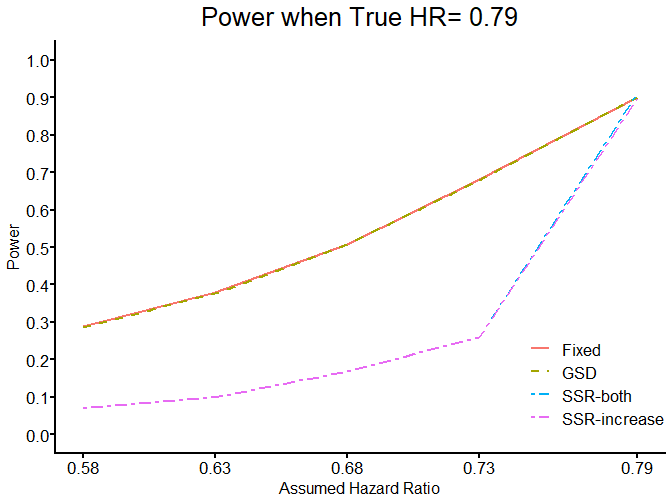
*

e)

c)

d)

a)

b)

*Figure SM14: Comparison of Group Sequential Design (GSD) and Sample Size Re-estimation (SSR, utilising 5,000 simulations and an optimisation prevention effect, HR=0.79) with O’Brien-Fleming boundaries against Fixed Randomised Controlled Trial for (a) expected number of events, (b) maximum number of events, (c) probability (Prob) of stopping for efficacy at the interim analysis (IA), (d) Prob of stopping for futility at the IA and (e) power, for five different prior HRs when the true HR=0.79, for power of 90%, with a single IA placed at 64% of the way through the trial.*

*
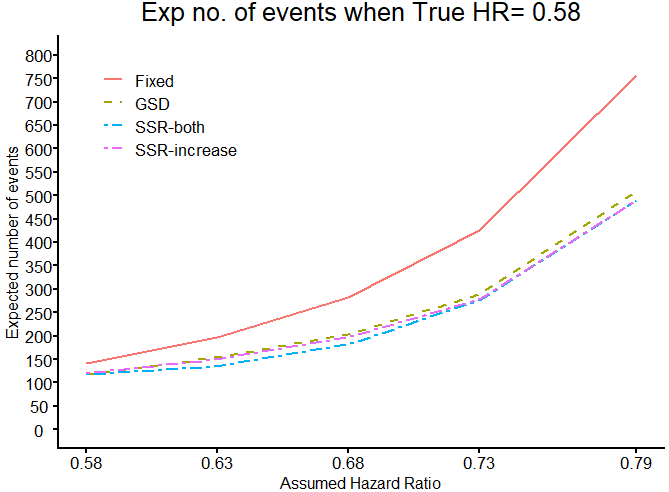

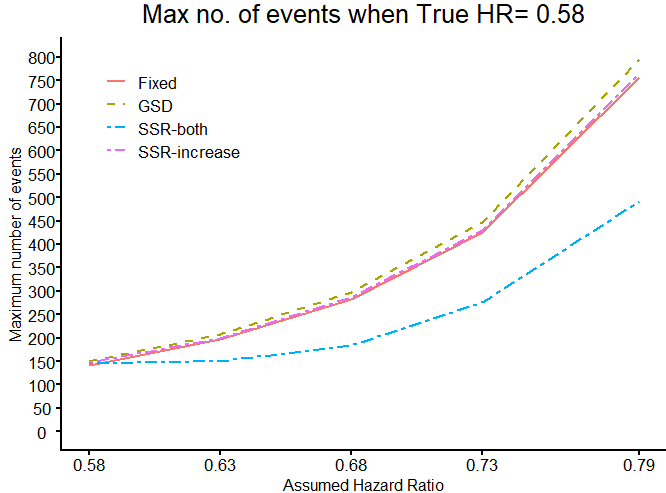

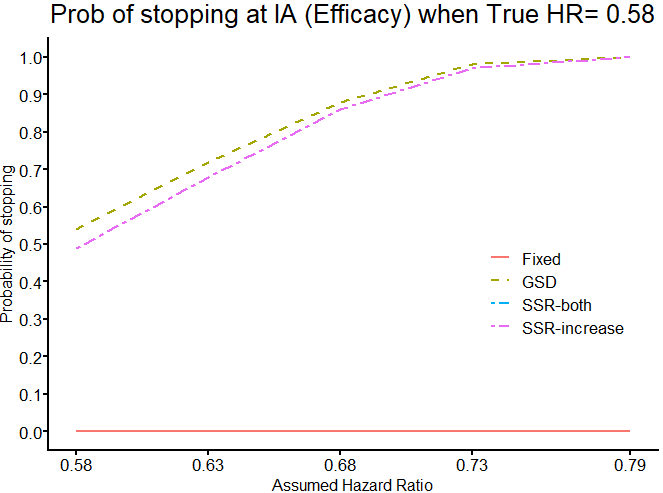

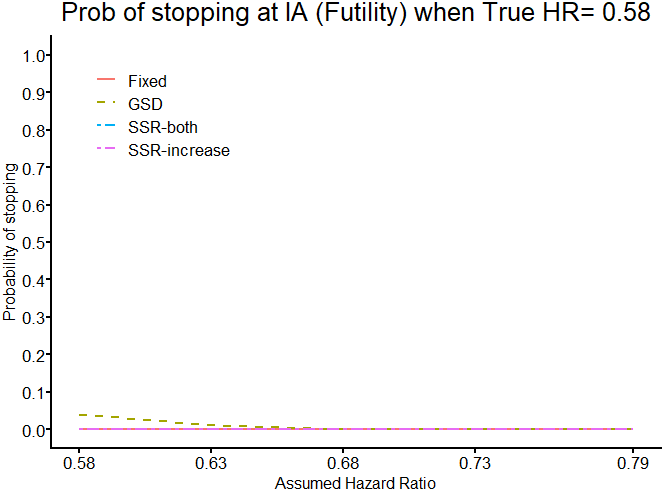

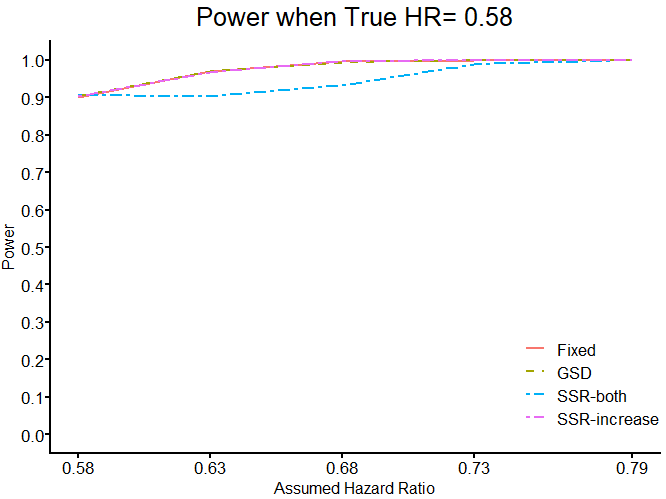
*

d)

e)

c)

b)

a)

*Figure SM15: Comparison of Group Sequential Design (GSD) and Sample Size Re-estimation (SSR, utilising 5,000 simulations and an optimisation prevention effect, HR=0.58) with O’Brien-Fleming boundaries against Fixed Randomised Controlled Trial for (a) expected number of events, (b) maximum number of events, (c) probability (Prob) of stopping for efficacy at the interim analysis (IA), (d) Prob of stopping for futility at the IA and (e) power, for five different prior HRs when the true HR=0.58, for power of 90%, with a single IA placed at 64% of the way through the trial.*
