# Supplementary material for: Benefits of group sequential design and sample size re-estimation for randomised controlled trials evaluating the prevention of ventilator-associated pneumonia: a simulation study informed by real world data
Source: BMC Med Res Methodol. 2025 Nov 12;25:254. doi: 10.1186/s12874-025-02681-4 (PMC12613641; doi:10.1186/s12874-025-02681-4)
Supplement: Supplementary file 2 — Additional file 2: Benefits of group sequential design and sample size re-estimation for RCTs evaluating the prevention of ventilator-associated pneumonia: A simulation study informed by real world data. Document containing the R code which was used to produce the results in this manuscript. [file 12874_2025_2681_MOESM2_ESM.docx]

**Additional Material 2 for *Benefits of group sequential design and sample size re-estimation for RCTs evaluating the prevention of ventilator-associated pneumonia: A simulation study informed by real world data***

Please find here, R code which was used to produce the results in this manuscript.

#Load Packages

library(rpact)

library(strucchange)

library(MASS)

library(nlme)

library(lme4)

library(glmmTMB)

library(tcltk)

library(zoo)

library(plyr)

library(ggplot2)

library(multcomp)

library(mice)

library(cowplot)

library(gridExtra)

library(readxl)

library(pscl)

library(bbmle)

library(scales)

library(openxlsx)

library(lubridate)

library(cmprsk)

library(survminer)

library(epiR)

library(tidyr)

library(dplyr)

library(stringr)

library(tidytable)

library(ggpubr)

library(patchwork)

library(tibble)

##RECRUITMENT RATE

#Create data frame with recruitment date and cumulative frequency of patients recruited on each date #using HONEST-PREPS data

#df.recruit.vent <- as.data.frame(table(all.dates.vent$icu.adm.date))

#colnames(df.recruit.vent) <- c("Date", "n.recruit")

#df.recruit.vent$Date <- as.Date(df.recruit.vent$Date)

#df.recruit.vent$cumsum <- cumsum(df.recruit.vent$n.recruit)

#df.recruit.vent$diffdate <- df.recruit.vent$Date - df.recruit.vent$Date[1]

## fit null hypothesis model and all potential models with multiple breakpoints

#fm0 <- lm(cumsum ~ diffdate, data=df.recruit.vent)

#fm1 <- lm(cumsum ~ diffdate*breakfactor(bp.rec, breaks = 1), data=df.recruit.vent)

#fm2 <- lm(cumsum ~ diffdate*breakfactor(bp.rec, breaks = 2), data=df.recruit.vent)

#df.fm0.pred <- data.frame(Date=df.recruit.vent$Date, cumsum = predict(fm0, df.recruit.vent))

#df.fm1.pred <- data.frame(Date=df.recruit.vent$Date, cumsum = predict(fm1, df.recruit.vent))

#df.fm2.pred <- data.frame(Date=df.recruit.vent$Date, cumsum = predict(fm2, df.recruit.vent))

#df.recruit.vent.all <- rbind(cbind(df.recruit.vent[,c("Date", "cumsum")], group="Raw Data"),

# cbind(df.fm0.pred, group="0-breakpoint regression"),

# cbind(df.fm1.pred, group="1-breakpoint regression"),

# cbind(df.fm2.pred, group="2-breakpoint regression"))

#Plot all breakpoint models

#plot.recruit.vent.all <- ggplot(df.recruit.vent.all, aes(x=Date, y=cumsum, group=group, col=group)) +

# geom_hline(yintercept=seq(0,1250,250), col="grey80")+

# geom_line(size=1)+

# scale_x_date(name="Admission Dates", breaks=as.Date(c("2020-06-01", "2020-12-01",

# "2021-06-01", "2021-12-01",

# "2022-05-01")),

# limits=as.Date(c(min(df.recruit.vent.all$Date), max(df.recruit.vent.all$Date))),

# date_labels="%d%b%y")+

# scale_y_continuous(name="N patients enrolled (cumulative)", breaks=seq(0, 2000, 250)) +

# scale_colour_manual(values=c("purple", "dodgerblue", "red", "green", "black"))+

# theme_j()

#plot.recruit.vent.all

# Looking at the plot, the '0 breakpoints' describes the recruitment best,

# If we use 0 breakpoints then recruitment is:

#Rec<-max(df.recruit.vent$cumsum) #1096 patients

#Time<- max(df.recruit.vent$Date)-df.recruit.vent$Date[1] #715 days

#Time_month<-Time/(365.25/12) #23.49 months
#Time_month<-as.numeric(Time_month)
#Recrate<-round(Rec/Time_month) #47 patients per month

#Input average monthly recruitment rate

Rec_rate<-47

## Compute piecewise exponential distribution

#VAP cumulative incidence #using HONEST-PREPS data

#CIF_VAP_data<-inc.vap.fin

#CIF_VAP_data$Event<-inc.vap.fin$event.type

#CIF_VAP_data<-transform(CIF_VAP_data, Event= ifelse(event.type=="death", "Death", Event))

#CIF_VAP_data<-transform(CIF_VAP_data, Event= ifelse(event.type=="hapvap", "VAP", Event))

#CIF_VAP_data<-transform(CIF_VAP_data, Event= ifelse(event.type=="icu.dis", "Discharged from ICU", Event))

#CIF_VAP_data<-transform(CIF_VAP_data, Event= ifelse(event.type=="extub", "Extubation", Event))

#CIF_VAP_data<-transform(CIF_VAP_data, Event= ifelse(event.type=="0", 0, Event))

#Restrict CIF to 60 days

#df.cif.60 <- CIF_VAP_data

#df.cif.60[df.cif.60$event.time>60, "Event"] <- 0

#df.cif.60[df.cif.60$event.time>60, "event.time"] <- 60
#cif.60 <- cuminc(df.cif.60$event.time, df.cif.60$Event)

#df.cif.60.plot <- data.frame(time=cif.60$`1 VAP`$time, event=cif.60$`1 VAP`$est)

#Invert CIF to get survival function. Then compute LOESS to get smoothed values of survival function.

#pneu.surv <- data.frame(time=df.cif.60.plot$time, surv=1-df.cif.60.plot$event)

#cif1 <- loess(surv ~ time, data=pneu.surv, span=0.2)

#smoothed10 <- predict(cif1, data.frame(time = 0:58))

#cif.smooth <- unique(data.frame(time=0:58, surv=smoothed10))

# Based on smoothed survival function

# time points at which survival function is known (must include 0)

times <- c(0, 2, 5, 9, 12, 15, 18, 27, 58)

# Survival function at timepoints t #(based on cif.smooth, computed from HONEST-PREPS data)

#S <- cif.smooth[cif.smooth$time %in% times, "surv"]

#S[1] <- 1

S <- c(1, 0.9424742, 0.8902680, 0.8682283, 0.8613409, 0.8507803, 0.8484055, 0.8451096, 0.8422946) #Results from HONEST-PREPS

# Convert days to months. To make sure with rpact interpretation.

times <- times/(365.25/12)

#https://www.rpact.org/vignettes/planning/rpact_survival_examples/

lambda <- -diff(log(S))/diff(times)

# Define parameters for piecewise exponential distribution in the control arm

lambda.ctrl <- c(lambda, tail(lambda, n = 1))

# piecewise exponential distribution

tp <- seq(0, 58, by = 0.01)/(365.25/12)

pw.exp.distr <- 1 - getPiecewiseExponentialDistribution(time = tp,

piecewiseSurvivalTime = times,

piecewiseLambda = lambda.ctrl)

pneu.surv2 <- cbind(data.frame(time=tp, surv=pw.exp.distr), fun="PW EXP")

pneu.surv2$time <- ifelse(pneu.surv2$fun=="PW EXP", pneu.surv2$time,

pneu.surv2$time/(365.25/12))

# Determine hazards in control group at day 28

t <- 28

S.c <- subset(pneu.surv2, fun=="PW EXP" & time==t/(365.25/12))$surv

F.c <- 1 - S.c

(CI.cont<-F.c)

# Compute Cum Inc in investigational arm for certain reduction in VAP incidence

Reduction<-c(0.2, 0.25, 0.3, 0.35, 0.4, 0.45)

# Determine hazard in investigational group

(CI.inv<-F.c*(1-Reduction))

F.e <- (1-Reduction) * F.c

S.e <- 1 - F.e

# Compute hazard ratio

HR_vec.all <- round(log(S.e)/log(S.c), 2)

HR_vec<-HR_vec.all[1:5]

HR_tab<-cbind(Reduction*100, round(CI.inv, 3), HR_vec.all)

colnames(HR_tab)<-c("% Reduction in VAP", "Cum Inc in inv", "HR")

HR_tab<-as.data.frame(HR_tab)

HR_tab

#Design choices

# Superiority trial, one-sided

sided.test=1

# False positive rate = 2.5%

if (sided.test==1){

alph=0.025

} else if (sided.test==2) {

alph=0.05

}

# Investigate Power = 80% and 90%

Pow_vec=c(0.8, 0.9)

## Get sample size for Fixed design

count<-0

SS.fix_vec<- data.frame()

for (Pow in Pow_vec) {

for (hr in HR_vec.all) {

count<-count+1

ss.fixed <- getSampleSizeSurvival(sided = sided.test,

alpha = alph,

beta = 1-Pow,

piecewiseSurvivalTime = times,

lambda2 = lambda.ctrl,

hazardRatio = hr,

accrualTime = 0,

accrualIntensity = Rec_rate,

followUpTime = 1)

#Variables of interest

SS.fix_vec[count, "HR"] <- hr

SS.fix_vec[count, "Interim"] <- NA

SS.fix_vec[count, "E.events"] <- round(ss.fixed$eventsFixed, 3)

SS.fix_vec[count, "Max.events"] <- ceiling(ss.fixed$eventsFixed)

SS.fix_vec[count, "E.subjects"] <- round(ss.fixed$maxNumberOfSubjects, 3)

SS.fix_vec[count, "Max.subjects"] <- ceiling(ss.fixed$maxNumberOfSubjects)

SS.fix_vec[count, "E.SD"] <- round(ss.fixed$studyDuration, 1)

SS.fix_vec[count, "Prob.stop.Eff"] <- 0

SS.fix_vec[count, "Prob.stop.Fut"] <- 0

SS.fix_vec[count, "Prob.stop"] <- 0

SS.fix_vec[count, "Power"] <- Pow

}

}

## GROUP SEQUENTIAL DESIGN

## O'Brien-Fleming design with 1 interim analysis and 1 final analysis

# Vary timing of interim analysis

plot.df<- data.frame()

count<-0

for (Pow in Pow_vec){

for (hr.plot in HR_vec){

if (Pow==0.8){

Z=c(seq(0.1, 0.6, 0.1), c(0.63, 0.64, 0.65), seq(0.7, 0.9, 0.1))

} else {

Z=c(seq(0.1, 0.6, 0.1), c(0.63, 0.64, 0.65), seq(0.7, 0.9, 0.1))

}

for (i in Z){

count <- count+1

dGS <- getDesignGroupSequential(sided = sided.test,

alpha = alph,

beta = 1-Pow,

informationRates = c(i, 1),

typeOfDesign = "asOF",

typeBetaSpending = "bsOF")

ss.gsd <- getSampleSizeSurvival(design = dGS,

piecewiseSurvivalTime = times,

lambda2 = lambda.ctrl,

hazardRatio = hr.plot,

accrualTime = 0,

accrualIntensity = Rec_rate,

followUpTime = 1)

#Variables of interest

plot.df[count, "HR"] <- hr.plot

plot.df[count, "Interim"] <- i

plot.df[count, "E.events"] <- round(ss.gsd$expectedEventsH1, 3)

plot.df[count, "Max.events"] <- ceiling(ss.gsd$maxNumberOfEvents)

plot.df[count, "E.subjects"] <- round(ss.gsd$expectedNumberOfSubjectsH1, 3)

plot.df[count, "Max.subjects"] <- ceiling(ss.gsd$maxNumberOfSubjects)

plot.df[count, "E.SD"] <- round(ss.gsd$studyDuration, 1)

plot.df[count, "Prob.stop.Eff"] <- round(ss.gsd$rejectPerStage[1], 2)

plot.df[count, "Prob.stop.Fut"] <- round(ss.gsd$futilityPerStage[1], 2)

plot.df[count, "Prob.stop"] <- round(ss.gsd$earlyStop, 2)

plot.df[count, "Power"] <- round(sum(ss.gsd$rejectPerStage), 1)

}

}

}

L=length(HR_vec)

L2=length(Pow_vec)

Opt_IA<-matrix(, nrow = L, ncol = L2)

for (l2 in seq(1, L2, 1)){

for (l in seq(1, L, 1)){

#Find the IA with optimum expected events

Opt_IA[l, l2]<-plot.df$Interim[plot.df$E.events==min(plot.df$E.events[plot.df$HR==HR_vec[l] & plot.df$Power==Pow_vec[l2]]) & plot.df$HR==HR_vec[l] & plot.df$Power==Pow_vec[l2]]

}

}

## Pocock design with 1 interim analysis and 1 final analysis

# Vary timing of interim analysis

plot.df.Po<- data.frame()

count<-0

for (Pow in Pow_vec){

for (hr.plot in HR_vec){

if (Pow==0.8){

Z=c(seq(0.1, 0.4, 0.1), 0.47, 0.48, 0.49, seq(0.6, 0.9, 0.1))

} else {

Z=c(seq(0.1, 0.4, 0.1), 0.45, 0.46, 0.47, seq(0.5, 0.9, 0.1))

}

for (i in Z){

count <- count+1

dGS <- getDesignGroupSequential(sided = sided.test,

alpha = alph,

beta = 1-Pow,

informationRates = c(i, 1),

typeOfDesign = "asP",

typeBetaSpending = "bsP")

ss.gsd <- getSampleSizeSurvival(design = dGS,

piecewiseSurvivalTime = times,

lambda2 = lambda.ctrl,

hazardRatio = hr.plot,

accrualTime = 0,

accrualIntensity = Rec_rate,

followUpTime = 1)

#Variables of interest

plot.df.Po[count, "HR"] <- hr.plot

plot.df.Po[count, "Interim"] <- i

plot.df.Po[count, "E.events"] <- round(ss.gsd$expectedEventsH1, 3)

plot.df.Po[count, "Max.events"] <- ceiling(ss.gsd$maxNumberOfEvents)

plot.df.Po[count, "E.subjects"] <- round(ss.gsd$expectedNumberOfSubjectsH1, 3)

plot.df.Po[count, "Max.subjects"] <- ceiling(ss.gsd$maxNumberOfSubjects)

plot.df.Po[count, "E.SD"] <- round(ss.gsd$studyDuration, 1)

plot.df.Po[count, "Prob.stop.Eff"] <- round(ss.gsd$rejectPerStage[1], 2)

plot.df.Po[count, "Prob.stop.Fut"] <- round(ss.gsd$futilityPerStage[1], 2)

plot.df.Po[count, "Prob.stop"] <- round(ss.gsd$earlyStop, 2)

plot.df.Po[count, "Power"] <- round(sum(ss.gsd$rejectPerStage), 1)

}

}

}

## Changing number of IAs from 1-9. Use OBF boundaries.

# For each number of IA have them evenly spaced through the trial

plot.df.10IA<- data.frame()

count<-0

for (Pow in Pow_vec){

for (hr.plot in HR_vec){

for (I in seq(1, 9, 1)){

count <- count+1

IA_vec=round(seq(1/(I+1), 1, 1/(I+1)), 10)

dGS <- getDesignGroupSequential(sided = sided.test,

alpha = alph,

beta = 1-Pow,

informationRates = IA_vec,

typeOfDesign = "asOF",

typeBetaSpending = "bsOF")

ss.gsd <- getSampleSizeSurvival(design = dGS,

piecewiseSurvivalTime = times,

lambda2 = lambda.ctrl,

hazardRatio = hr.plot,

accrualTime = 0,

accrualIntensity = Rec_rate,

followUpTime = 1)

#Variables of interest

plot.df.10IA[count, "HR"] <- hr.plot

plot.df.10IA[count, "no.IAs"] <- I

plot.df.10IA[count, "E.events"] <- round(ss.gsd$expectedEventsH1, 3)

plot.df.10IA[count, "Max.events"] <- ceiling(ss.gsd$maxNumberOfEvents)

plot.df.10IA[count, "E.subjects"] <- round(ss.gsd$expectedNumberOfSubjectsH1, 3)

plot.df.10IA[count, "Max.subjects"] <- ceiling(ss.gsd$maxNumberOfSubjects)

plot.df.10IA[count, "E.SD"] <- round(ss.gsd$studyDuration, 1)

plot.df.10IA[count, "Prob.stop.Eff"] <- round(sum(ss.gsd$rejectPerStage[1:I]), 2)

plot.df.10IA[count, "Prob.stop.Fut"] <- round(sum(ss.gsd$futilityPerStage[1:I]), 2)

plot.df.10IA[count, "Prob.stop"] <- round(ss.gsd$earlyStop, 2)

plot.df.10IA[count, "Power"] <- round(sum(ss.gsd$rejectPerStage), 1)

}

}

}

# SAMPLE SIZE RE-ESTIMATION

# Demonstrate use of SSR

set.seed(50)

# Function to perform SSR

E_events <- function(n.max, HR, Rec_rate, times, lambda.ctrl, sided.test, alph, Pow2,

n.interim, n.total, n.increase, max.subj, simulation.tot, seed) {

#Input Variables

#n.max: max number of events can increase to, to produce a conditional power= Pow2%

#HR: Hazard Ratio

#Rec_rate: Recruitment Rate

#times: timepoints

#lambda.ctrl: Hazard rate in control treatment at timepoints t

#sided.test: 1 or 2 sided test?

#alph: type I error

#Pow2: Conditional power want design to reach after SSR

#n.interim: number of events at interim analysis

#n.total: initial total number of events (from GSD)

#max.subj: Max total number of patients

dIN <- getDesignInverseNormal(sided=sided.test, alpha=alph, beta=1-Pow2,

informationRates = c(n.interim/n.total, 1),

typeOfDesign="asOF")

ss.ssr <- getSimulationSurvival(dIN, lambda2 = lambda.ctrl,

hazardRatio = HR,

piecewiseSurvivalTime = times,

accrualTime = 0,

accrualIntensity = Rec_rate,

maxNumberOfSubjects =

ceiling(max.subj+8000),

plannedEvents = c(n.interim, n.total),

directionUpper = FALSE,

maxNumberOfIterations = simulation.tot,

conditionalPower = Pow2,

minNumberOfEventsPerStage = c(n.interim,

n.increase),

maxNumberOfEventsPerStage = c(n.interim,

n.max-n.interim),

seed = seed)

#Variables of interest

plot.df.opt<-list(HR,

n.interim,

n.total,

n.max,

round(ss.ssr$expectedNumberOfEvents, 3),

ceiling(sum(ss.ssr$eventsPerStage)),

round(ss.ssr$expectedNumberOfSubjects, 3),

ceiling(ss.ssr$numberOfSubjects[2]),

round(ss.ssr$studyDuration, 1),

round(ss.ssr$rejectPerStage[1], 2),

round(ss.ssr$futilityStop, 2),

round(ss.ssr$earlyStop, 2),

ss.ssr$overallReject)

return(plot.df.opt)

}

#Function to optimise the total maximum number of events to allow conditional power to equal target

objfun <- function(x, HR, Rec_rate, times, lambda.ctrl, sided.test, alph, Pow2, n.interim, n.total, n.increase, max.subj, target, simulation.tot, seed) {

sqrt((E_events(x, HR, Rec_rate, times, lambda.ctrl, sided.test, alph, Pow2, n.interim, n.total, n.increase, max.subj, simulation.tot, seed)[[13]]-target)^2) }

#Constants to input into optimisation function

Pow2=0.8 #desired power

int=Opt_IA[1, 1] #Optimal placement of IA from GSD

target=Pow2 #desired power after sample size re-estimation

Pow1=Pow2 #desired power in initial GSD trial

HR.true=0.68 #True HR

HR.optimise=0.68 #Optimisation HR

HR=0.63 #Assumed HR

seed.opt=1234 #Simulation seed for optimisation function

seed.sim=123 #simulation seed for simulaiton

#Initial GSD trial sample size calculation

design <- getDesignGroupSequential(sided=sided.test, alpha=alph, beta=1-Pow1,

informationRates = c(int, 1),

typeOfDesign = "asOF")

ss.gsd <- getSampleSizeSurvival(design = design,

piecewiseSurvivalTime = times,

lambda2 = lambda.ctrl,

hazardRatio = HR,

accrualTime = 0,

accrualIntensity = Rec_rate,

followUpTime = 1)

#Find interim SS and min total SS

(interim.subj<-ceiling(ss.gsd$numberOfSubjects[1]))

(max.subj<-ceiling(ss.gsd$maxNumberOfSubjects))

#Find min total events and interim events

(n.total <- ceiling(ss.gsd$eventsPerStage[2]))

(n.interim <- ceiling(ss.gsd$eventsPerStage[1]))

n.increase.1<-n.total-n.interim #Only allow number of events to increase at IA

#Maximum possible number of events allowed to increase to (double number of events from fixed RCT)

Max.EVENTS<-2*SS.fix_vec$Max.events[SS.fix_vec$HR==HR & SS.fix_vec$Power==Pow2]

#Use fewer simulations for optimisation to save on computational time

simulation.opt<-2000 #Total simulations for optimisation

simulation.sim<-10000 #Total simulations for running trial

# Find optimal total maximum number of events to allow conditional power to equal target

res <- optimize(function(x, HR.optimise, Rec_rate, times, lambda.ctrl, sided.test, alph, Pow2, n.interim, n.total, n.increase.1, max.subj, target, simulation.opt , seed.opt)

objfun(x, HR.optimise, Rec_rate, times, lambda.ctrl, sided.test, alph, Pow2, n.interim, n.total, n.increase.1, max.subj, target, simulation.opt , seed.opt),

interval = c(n.total+1, Max.EVENTS), HR.optimise, Rec_rate, times, lambda.ctrl, sided.test, alph, Pow2, n.interim, n.total, n.increase.1, max.subj, target, simulation.opt , seed.opt)

res.opt<-ceiling(res$minimum)

# Use optimal total max number of events to perform SAMPLE SIZE RE-ESTIMATION

dIN <- getDesignInverseNormal(sided = sided.test,

alpha = alph,

beta = 1-Pow1,

informationRates = c(n.interim/n.total, 1),

typeOfDesign="asOF" ,

futilityBounds = 0,

bindingFutility = FALSE)

ss.ssr.68 <- getSimulationSurvival(dIN, lambda2 = lambda.ctrl, hazardRatio = HR.true,

piecewiseSurvivalTime = times,

accrualTime = 0,

accrualIntensity = Rec_rate,

maxNumberOfSubjects =

max.subj+8000,

plannedEvents = c(n.interim, n.total),

directionUpper = FALSE,

maxNumberOfIterations = simulation.sim,

longTimeSimulationAllowed = TRUE,

conditionalPower = target,

minNumberOfEventsPerStage = c(n.interim, n.increase.1),

maxNumberOfEventsPerStage = c(n.interim, res.opt-n.interim),

seed = seed.sim)

ss.ssr.68$hazardRatio #HR

ss.ssr.68$overallReject #Power

ss.ssr.68$earlyStop #Prob of stopping early

ss.ssr.68$expectedNumberOfEvents #Expected number of events

ceiling(sum(ss.ssr.68$eventsPerStage)) #Mean maximum number of events across simulations

res.opt #Total max number of events

ss.ssr.68$expectedNumberOfSubjects #Expected number of subjects

ceiling(ss.ssr.68$numberOfSubjects[2]) #Mean maximum number of subjects

summary(ss.ssr.68)

# Create histogram to display number of events in each iteration of the SSR simulation

df <- getData.SimulationResults(ss.ssr.68)

overallSampleSizes <- sapply(1:10000, function(i) {

sum(df[df$iterationNumber == i,]$eventsPerStage)

})

SSR.hist<-hist(overallSampleSizes, main = "", xlab = "Required number of events", breaks=100, xlim=c(90, 300), ylim=c(0, 4000))

Tab<-table(overallSampleSizes)

Tab<-as.data.frame(Tab)

Tab$overallSampleSizes<-ordered(Tab$overallSampleSizes)

Tab[Tab$overallSampleSizes==n.interim, 2]/10000 ## 96 events (26.9% of the time, stop at interim)

Tab[Tab$overallSampleSizes==n.total, 2]/10000 ## 149 events (20.1% of the time, stop with min # of events)

sum(Tab[Tab$overallSampleSizes==res.opt, 2])/10000 ## 287 events (32.7% of the time, stop with max # of events)

## Remaining 20.3% are between 150 and 286. More or less evenly spaced

(10000-(Tab[Tab$overallSampleSizes==n.interim, 2]+Tab[Tab$overallSampleSizes==n.total, 2]+sum(Tab[Tab$overallSampleSizes==res.opt, 2])))/10000

#At max number of events, proportion which did/didn’t reach significance

#1 if null hypothesis can be rejected, 0 otherwise.

aaa <- subset(df, stageNumber==2 & eventsPerStage>=(res.opt-n.interim))

table(aaa$rejectPerStage)

#If study does not increase number of events at n=149, proportion which did/didn’t reach significance

bbb <- subset(df, stageNumber==2 & eventsPerStage==(n.total-n.interim))

table(bbb$rejectPerStage)

#Number of events increased between 1 and 135 patients, proportion which did/didn’t reach significance

ddd <- subset(df, stageNumber==2 & !(eventsPerStage %in% c((n.total-n.interim), (res.opt-n.interim))))

table(ddd$rejectPerStage)

#Study stopped at interim n=96, proportion which efficacy reached

ccc <- subset(df, stageNumber==1 & rejectPerStage==1)

table(ccc$rejectPerStage)

# Study stopped at interim n=96, proportion which futility reached

ccc.2 <- subset(df, stageNumber==1 & futilityPerStage==1)

table(ccc.2$futilityPerStage)

#Power is

(table(ccc$rejectPerStage)+table(ddd$rejectPerStage)[2]+table(bbb$rejectPerStage)[2]+table(aaa$rejectPerStage)[2])/10000

## SAMPLE SIZE RE-ESTIMATION

#Simulation function

Exp_events <- function(n.max, HR, Rec_rate, times, lambda.ctrl, sided.test, alph, Pow2,

n.interim, n.total, n.increase, max.subj, simulation.tot, seed) {

#Input Variables

#n.max: max sample size increase to get an overall Pow2% power

#HR: Hazard Ratio

#Rec_rate: Recruitment Rate

#times: timepoints

#lambda.ctrl: Hazard rate in control treatment at timepoints t

#sided.test: 1 or 2 sided test?

#alph: type I error

#Pow2: Conditional power want design to reach after SS re-estimation

#n.interim: interim analysis

#n.total: initial total sample size (from fixed design)

#max.subj:

#Output Variables

#Function returns a list consisting of:

#HR

#IA

#Initial total Sample Size

#New Maximum total sample size

#Expected events

#Average Maximum events

#Max Maximum events

#Expected Sample Size

#Maximum Sample Size

#Expected Study Duration

#Maximum Study Duration

#Probability of early stop

#Conditional Power

dIN <- getDesignInverseNormal(sided=sided.test, alpha=alph, beta=1-Pow2,

informationRates = c(n.interim/n.total, 1),

typeOfDesign="asOF",

futilityBounds = 0,

bindingFutility = FALSE)

ss.ssr <- getSimulationSurvival(dIN, lambda2 = lambda.ctrl,

hazardRatio = HR,

piecewiseSurvivalTime = times,

accrualTime = 0,

accrualIntensity = Rec_rate,

maxNumberOfSubjects =

ceiling(max.subj+8000),

plannedEvents = c(n.interim, n.total),

directionUpper = FALSE,

maxNumberOfIterations = simulation.tot,

conditionalPower = Pow2,

minNumberOfEventsPerStage = c(n.interim,

n.increase),

maxNumberOfEventsPerStage = c(n.interim,

n.max-n.interim),

seed = seed)

#Variables of interest

plot.df.opt<-list(HR,

n.interim,

n.total,

n.max,

round(ss.ssr$expectedNumberOfEvents, 3),

ceiling(sum(ss.ssr$eventsPerStage)),

round(ss.ssr$expectedNumberOfSubjects, 3),

ceiling(ss.ssr$numberOfSubjects[2]),

round(ss.ssr$studyDuration, 1),

round(ss.ssr$rejectPerStage[1], 2),

round(ss.ssr$futilityStop, 2),

round(ss.ssr$earlyStop, 2),

ss.ssr$overallReject)

return(plot.df.opt)

}

# Use Assumed HR with prevention effect 5% larger than true HR

count<-0

res_vec_2.SSR<-data.frame()

res_vec_both.SSR<-data.frame()

Pow2=0.8 #Power

int=Opt_IA[1,1] #Optimal IA placement from GSD

target=Pow2

Pow1=Pow2

HR_vec.2=c(0.53, 0.63, 0.73) # Assumed HR with prevention effect 5% larger than true HR

for (HR.true in c(0.58, 0.68, 0.79)) {

set.seed(50)

count<-count+1

HR=HR_vec.2[count]

HR.optimise= HR.true

print(HR)

design <- getDesignGroupSequential(sided=sided.test, alpha=alph, beta=1-Pow1,

informationRates = c(int, 1),

typeOfDesign = "asOF")

ss.gsd <- getSampleSizeSurvival(design = design,

piecewiseSurvivalTime = times,

lambda2 = lambda.ctrl,

hazardRatio = HR,

accrualTime = 0,

accrualIntensity = Rec_rate,

followUpTime = 1)

#Find interim SS and min total SS

(interim.subj<-ceiling(ss.gsd$numberOfSubjects[1]))

(max.subj<-ceiling(ss.gsd$maxNumberOfSubjects))

#Find min total events and interim events

(n.total <- ceiling(ss.gsd$eventsPerStage[2]))

(n.interim <- ceiling(ss.gsd$eventsPerStage[1]))

n.increase.1<-n.total-n.interim #Only allow number of events to increase at IA

n.increase.2<-1 #Allow number of events to also decrease at IA

#Maximum possible number of events allowed to increase to (double number of events from fixed RCT)

Max.EVENTS<-2*SS.fix_vec$Max.events[SS.fix_vec$HR==HR & SS.fix_vec$Power==Pow2]

#Find optimal total maximum number of events, allowing SSR to increase only

res <- optimize(function(x, HR.optimise, Rec_rate, times, lambda.ctrl, sided.test, alph, Pow2, n.interim, n.total, n.increase.1, max.subj, target, simulation.opt , seed.opt)

objfun(x, HR.optimise, Rec_rate, times, lambda.ctrl, sided.test, alph, Pow2, n.interim, n.total, n.increase.1, max.subj, target, simulation.opt , seed.opt),

interval = c(n.total+1, Max.EVENTS), HR.optimise, Rec_rate, times, lambda.ctrl, sided.test, alph, Pow2, n.interim, n.total, n.increase.1, max.subj, target, simulation.opt , seed.opt)

res.opt<-ceiling(res$minimum)

#Find sample size for SSR-increase using optimal total maximum number of events

res_vec_2.SSR[count, "Prior.HR"]<-HR

res_vec_2.SSR[count, 2:14]<-Exp_events(res.opt, HR.true, Rec_rate, times, lambda.ctrl, sided.test, alph, Pow2, n.interim, n.total, n.increase.1, max.subj, simulation.sim , seed.sim)

res_vec_2.SSR[count, "Target.Power"] <- Pow2

#Find optimal total maximum number of events, allowing SSR to increase or decrease

res <- optimize(function(x, HR.optimise, Rec_rate, times, lambda.ctrl, sided.test, alph, Pow2, n.interim, n.total, n.increase.2, max.subj, target, simulation.opt , seed.opt)

objfun(x, HR.optimise, Rec_rate, times, lambda.ctrl, sided.test, alph, Pow2, n.interim, n.total, n.increase.2, max.subj, target, simulation.opt , seed.opt),

interval = c(n.interim+1, Max.EVENTS), HR.optimise, Rec_rate, times, lambda.ctrl, sided.test, alph, Pow2, n.interim, n.total, n.increase.2, max.subj, target, simulation.opt , seed.opt)

res.opt<-ceiling(res$minimum)

#Find sample size for SSR-both using optimal total maximum number of events

res_vec_both.SSR[count, "Prior.HR"]<-HR

res_vec_both.SSR[count, 2:14]<-Exp_events(res.opt, HR.true, Rec_rate, times, lambda.ctrl, sided.test, alph, Pow2, n.interim, n.total, n.increase.2, max.subj, simulation.sim, seed.sim)

res_vec_both.SSR[count, "Target.Power"] <- Pow2

if (res_vec_2.SSR[count, 14]<(Pow2-0.02)){

#If conditional power using optimal total maximum number of events does not reach desired power, stop trial at IA for futility

ssr.stop<-Exp_events((n.interim+1), HR.true, Rec_rate, times, lambda.ctrl, sided.test, alph, Pow2, n.interim, n.total, 1, max.subj, simulation.sim , seed.sim)

res_vec_2.SSR[count, 1:15]<-c(HR,

HR.true,

n.interim,

n.total,

n.interim,

n.interim,

n.interim,

ssr.stop[8],

ssr.stop[8],

ssr.stop[10],

ssr.stop[11],

1-as.numeric(ssr.stop[11]),

1.00,

ssr.stop[11],

Pow2)

}

if (res_vec_both.SSR[count, 14]<(Pow2-0.02)){

#If conditional power using optimal total maximum number of events does not reach desired power, stop trial at IA for futility

ssr.stop<-Exp_events((n.interim+1), HR.true, Rec_rate, times, lambda.ctrl, sided.test, alph, Pow2, n.interim, n.total, 1, max.subj, simulation.sim , seed.sim)

res_vec_both.SSR[count, 1:15]<-c(HR,

HR.true,

n.interim,

n.total,

n.interim,

n.interim,

n.interim,

ssr.stop[8],

ssr.stop[8],

ssr.stop[10],

ssr.stop[11],

1-as.numeric(ssr.stop[11]),

1.00,

ssr.stop[11],

Pow2)

}

}

colnames(res_vec_2.SSR)<-c("Prior.HR",

"True.HR",

"n.interim",

"n.total",

"n.max",

"E.events",

"Max.events",

"E.subjects",

"Max.subjects",

"E.SD",

"Prob.stop.Eff",

"Prob.stop.Fut",

"Prob.stop",

"Power",

"Target.Power")

res_vec_2.SSR

colnames(res_vec_both.SSR)<-c("Prior.HR",

"True.HR",

"n.interim",

"n.total",

"n.max",

"E.events",

"Max.events",

"E.subjects",

"Max.subjects",

"E.SD",

"Prob.stop.Eff",

"Prob.stop.Fut",

"Prob.stop",

"Power",

"Target.Power")

res_vec_both.SSR

## Compare Fixed, GSD, and SSR when assumed HR is incorrect

# Define true HR

HR.true.vec<-c(0.58, 0.68, 0.79, 1)

# Fixed RCT where Assumed HR does not equal true HR

count<-0

SS.fix_vec.inc<- data.frame()

for (Pow in Pow_vec) {

for (HR.true in HR.true.vec) {

for (hr in HR_vec) {

count<-count+1

ss.fixed.inc <- getSampleSizeSurvival(sided = sided.test,

alpha = alph,

beta = 1-Pow,

piecewiseSurvivalTime = times,

lambda2 = lambda.ctrl,

hazardRatio = hr,

accrualTime = 0,

accrualIntensity = Rec_rate,

followUpTime = 1)

ss.fixed <- getPowerSurvival(piecewiseSurvivalTime = times,

lambda2 = lambda.ctrl,

directionUpper = FALSE,

hazardRatio = HR.true,

accrualTime = 0,

accrualIntensity = Rec_rate,

maxNumberOfEvents = ceiling(ss.fixed.inc$eventsFixed),

maxNumberOfSubjects = ceiling(ss.fixed.inc$maxNumberOfSubjects))

#Variables of interest

SS.fix_vec.inc[count, "True.HR"] <- HR.true

SS.fix_vec.inc[count, "Prior.HR"] <- hr

SS.fix_vec.inc[count, "Interim"] <- NA

SS.fix_vec.inc[count, "E.events"] <- round(ss.fixed$expectedNumberOfEvents, 3)

SS.fix_vec.inc[count, "Max.events"] <- ceiling(ss.fixed$expectedNumberOfEvents)

SS.fix_vec.inc[count, "n.max"] <- ceiling(ss.fixed$expectedNumberOfEvents)

SS.fix_vec.inc[count, "E.subjects"] <- round(ss.fixed$expectedNumberOfSubjects, 3)

SS.fix_vec.inc[count, "Max.subjects"] <- ceiling(ss.fixed$expectedNumberOfSubjects)

SS.fix_vec.inc[count, "E.SD"] <- round(ss.fixed$studyDuration, 1)

SS.fix_vec.inc[count, "Prob.stop.Eff"] <- 0

SS.fix_vec.inc[count, "Prob.stop.Fut"] <- 0

SS.fix_vec.inc[count, "Prob.stop"] <- 0

SS.fix_vec.inc[count, "Power"] <- round(ss.fixed$overallReject, 3)

SS.fix_vec.inc[count, "Target.Power"] <- Pow

}

}

}

SS.fix_vec.inc

# GSD where Assumed HR does not equal true HR

count<-0

SS.gsd_vec<- data.frame()

for (Pow in Pow_vec) {

if (Pow==0.8){

int=Opt_IA[1, 1]

} else {

int=Opt_IA[1, 2]

}

for (HR.true in HR.true.vec) {

for (hr in HR_vec) {

count<-count+1

design <- getDesignGroupSequential(sided=sided.test, alpha=alph, beta=1-Pow,

informationRates = c(int, 1),

typeOfDesign = "asOF",

typeBetaSpending = "bsOF")

ss.gsd.inc <- getSampleSizeSurvival(design = design,

piecewiseSurvivalTime = times,

lambda2 = lambda.ctrl,

hazardRatio = hr,

accrualTime = 0,

accrualIntensity = Rec_rate,

followUpTime = 1)

ss.gsd <- getPowerSurvival(design = design,

piecewiseSurvivalTime = times,

lambda2 = lambda.ctrl,

directionUpper = FALSE,

hazardRatio = HR.true,

accrualTime = 0,

accrualIntensity = Rec_rate,

maxNumberOfEvents = ceiling(ss.gsd.inc$maxNumberOfEvents),

maxNumberOfSubjects = ceiling(ss.gsd.inc$maxNumberOfSubjects))

#Variables of interest

SS.gsd_vec[count, "True.HR"] <- HR.true

SS.gsd_vec[count, "Prior.HR"] <- hr

SS.gsd_vec[count, "Interim"] <- int

SS.gsd_vec[count, "E.events"] <- round(ss.gsd$expectedNumberOfEvents, 3)

SS.gsd_vec[count, "Max.events"] <- ceiling(ss.gsd$maxNumberOfEvents)

SS.gsd_vec[count, "n.max"] <- ceiling(ss.gsd$maxNumberOfEvents)

SS.gsd_vec[count, "E.subjects"] <- round(ss.gsd$expectedNumberOfSubjects, 3)

SS.gsd_vec[count, "Max.subjects"] <- ceiling(ss.gsd$maxNumberOfSubjects)

SS.gsd_vec[count, "E.SD"] <- round(ss.gsd$studyDuration, 1)

SS.gsd_vec[count, "Prob.stop.Eff"] <- round(ss.gsd$rejectPerStage[1], 2)

SS.gsd_vec[count, "Prob.stop.Fut"] <- round(ss.gsd$futilityStop, 2)

SS.gsd_vec[count, "Prob.stop"] <- round(ss.gsd$earlyStop, 2)

SS.gsd_vec[count, "Power"] <- round(ss.gsd$overallReject, 3)

SS.gsd_vec[count, "Target.Power"] <- Pow

}

}

}

SS.gsd_vec

# SSR where Assumed HR does not equal true HR

#Function to track sample size for SSR design

E_events <- function(n.max, HR, Rec_rate, times, lambda.ctrl, sided.test, alph, Pow2,

n.interim, n.total, n.increase, max.subj, simulation.tot, seed) {

#Input Variables

#n.max: max number of events can increase to, to produce a conditional power= Pow2%

#HR: Hazard Ratio

#Rec_rate: Recruitment Rate

#times: timepoints

#lambda.ctrl: Hazard rate in control treatment at timepoints t

#sided.test: 1 or 2 sided test?

#alph: type I error

#Pow2: Conditional power want design to reach after SSR

#n.interim: number of events at interim analysis

#n.total: initial total number of events (from GSD)

#max.subj: Max total number of patients

dIN <- getDesignInverseNormal(sided=sided.test, alpha=alph, beta=1-Pow2,

informationRates = c(n.interim/n.total, 1),

typeOfDesign="asOF")

ss.ssr <- getSimulationSurvival(dIN, lambda2 = lambda.ctrl,

hazardRatio = HR,

piecewiseSurvivalTime = times,

accrualTime = 0,

accrualIntensity = Rec_rate,

maxNumberOfSubjects =

ceiling(max.subj+8000),

plannedEvents = c(n.interim, n.total),

directionUpper = FALSE,

maxNumberOfIterations = simulation.tot,

conditionalPower = Pow2,

minNumberOfEventsPerStage = c(n.interim,

n.increase),

maxNumberOfEventsPerStage = c(n.interim,

n.max-n.interim),

seed = seed)

#Variables of interest

plot.df.opt<-list(HR,

n.interim,

n.total,

n.max,

round(ss.ssr$expectedNumberOfEvents, 3),

ceiling(sum(ss.ssr$eventsPerStage)),

round(ss.ssr$expectedNumberOfSubjects, 3),

ceiling(ss.ssr$numberOfSubjects[1]),

ceiling(ss.ssr$numberOfSubjects[2]),

round(ss.ssr$studyDuration, 1),

round(ss.ssr$rejectPerStage[1], 2),

round(ss.ssr$futilityStop, 2),

round(ss.ssr$earlyStop, 2),

ss.ssr$overallReject)

return(plot.df.opt)

}

#Function to optimise the total maximum number of events to allow conditional power to equal target

objfun <- function(x, HR, Rec_rate, times, lambda.ctrl, sided.test, alph, Pow2, n.interim, n.total, n.increase, max.subj, target, simulation.tot, seed) {

sqrt((E_events(x, HR, Rec_rate, times, lambda.ctrl, sided.test, alph, Pow2, n.interim, n.total, n.increase, max.subj, simulation.tot, seed)[[14]]-target)^2) }

Exp_events <- function(n.max, HR, Rec_rate, times, lambda.ctrl, sided.test, alph, Pow2,

n.interim, n.total, n.increase, max.subj, simulation.tot, seed) {

#Input Variables

#n.max: max sample size increase to get an overall Pow2% power

#HR: Hazard Ratio

#Rec_rate: Recruitment Rate

#times: timepoints

#lambda.ctrl: Hazard rate in control treatment at timepoints t

#sided.test: 1 or 2 sided test?

#alph: type I error

#Pow2: Conditional power want design to reach after SS re-estimation

#n.interim: interim analysis

#n.total: initial total sample size (from fixed design)

#max.subj:

#Output Variables

#Function returns a list consisting of:

#HR

#IA

#Initial total Sample Size

#New Maximum total sample size

#Expected events

#Average Maximum events

#Max Maximum events

#Expected Sample Size

#Maximum Sample Size

#Expected Study Duration

#Maximum Study Duration

#Probability of early stop

#Conditional Power

dIN <- getDesignInverseNormal(sided=sided.test, alpha=alph, beta=1-Pow2,

informationRates = c(n.interim/n.total, 1),

typeOfDesign="asOF",

futilityBounds = 0,

bindingFutility = FALSE)

ss.ssr <- getSimulationSurvival(dIN, lambda2 = lambda.ctrl,

hazardRatio = HR,

piecewiseSurvivalTime = times,

accrualTime = 0,

accrualIntensity = Rec_rate,

maxNumberOfSubjects =

ceiling(max.subj+8000),

plannedEvents = c(n.interim, n.total),

directionUpper = FALSE,

maxNumberOfIterations = simulation.tot,

conditionalPower = Pow2,

minNumberOfEventsPerStage = c(n.interim,

n.increase),

maxNumberOfEventsPerStage = c(n.interim,

n.max-n.interim),

seed = seed)

#Variables of interest

plot.df.opt<-list(HR,

n.interim,

n.total,

n.max,

round(ss.ssr$expectedNumberOfEvents, 3),

ceiling(sum(ss.ssr$eventsPerStage)),

round(ss.ssr$expectedNumberOfSubjects, 3),

ceiling(ss.ssr$numberOfSubjects[1]),

ceiling(ss.ssr$numberOfSubjects[2]),

round(ss.ssr$studyDuration, 1),

round(ss.ssr$rejectPerStage[1], 2),

round(ss.ssr$futilityStop, 2),

round(ss.ssr$earlyStop, 2),

ss.ssr$overallReject)

return(plot.df.opt)

}

#Use fewer simulations for optimisation to save on computational time

simulation.opt<-2000 #Total simulations for optimisation

simulation.sim<-5000 #Total simulations for running trial

count<-0

HR.true.vec<-c(0.58, 0.68, 0.79)

res_vec_2<-data.frame()

res_vec_2A<-data.frame()

for (Pow2 in Pow_vec) {

if (Pow2==0.8){

int=Opt_IA[1, 1] #Optimal placement of IA from GSD

} else {

int=Opt_IA[1, 2]

}

target=Pow2

Pow1=Pow2

for (HR.true in HR.true.vec) {

HR.optimise=HR.true

for (HR in HR_vec){

print(HR)

count<-count+1

design <- getDesignGroupSequential(sided=sided.test, alpha=alph, beta=1-Pow1,

informationRates = c(int, 1),

typeOfDesign = "asOF")

ss.gsd <- getSampleSizeSurvival(design = design,

piecewiseSurvivalTime = times,

lambda2 = lambda.ctrl,

hazardRatio = HR,

accrualTime = 0,

accrualIntensity = Rec_rate,

followUpTime = 1)

#Find interim SS and min total SS

(interim.subj<-ceiling(ss.gsd$numberOfSubjects[1]))

(max.subj<-ceiling(ss.gsd$maxNumberOfSubjects))

#Find min total events and interim events

(n.total <- ceiling(ss.gsd$eventsPerStage[2]))

(n.interim <- ceiling(ss.gsd$eventsPerStage[1]))

n.increase.1<-n.total-n.interim #Only allow number of events to increase at IA

n.increase.2<-1 #Allow number of events to also decrease at IA

#Maximum possible number of events allowed to increase to (double number of events from fixed RCT)

Max.EVENTS<-2*SS.fix_vec$Max.events[SS.fix_vec$HR==HR & SS.fix_vec$Power==Pow2]

#Simulate trial for pre-planned minimum number of events (with no sample size recalculation)

ssr<-Exp_events(n.total, HR.optimise, Rec_rate, times, lambda.ctrl, sided.test, alph, Pow2, n.interim, n.total, n.increase.1, max.subj, simulation.sim, seed.sim)

if (ssr[14]>Pow2){

#If conditional power is larger than target, use pre-planned minimum number of events

res.opt<- n.total

} else {

#If conditional power is not larger than target

#Find optimal total maximum number of events, allowing SSR to increase only

res <- optimize(function(x, HR.optimise, Rec_rate, times, lambda.ctrl, sided.test, alph, Pow2, n.interim, n.total, n.increase.1, max.subj, target, simulation.opt, seed.opt)

objfun(x, HR.optimise, Rec_rate, times, lambda.ctrl, sided.test, alph, Pow2, n.interim, n.total, n.increase.1, max.subj, target, simulation.opt, seed.opt),

interval = c(n.total+1, Max.EVENTS), HR.optimise, Rec_rate, times, lambda.ctrl, sided.test, alph, Pow2, n.interim, n.total, n.increase.1, max.subj, target, simulation.opt, seed.opt)

res.opt<-ceiling(res$minimum)

#Simulate trial for pre-planned minimum number of events (with no sample size recalculation)

ssr<-Exp_events(n.total, HR.optimise, Rec_rate, times, lambda.ctrl, sided.test, alph, Pow2, n.interim, n.total, n.increase.1, max.subj, simulation.sim, seed.sim)

}

if (res.opt>Max.EVENTS | ssr[14]<(Pow2-0.02)){

#If conditional power using optimal total maximum number of events does not reach desired power, stop trial at IA for futility

ssr.stop<-Exp_events((n.interim+1), HR.true, Rec_rate, times, lambda.ctrl, sided.test, alph, Pow2, n.interim, n.total, 1, max.subj, simulation.sim, seed.sim)

res_vec_2[count, 1:16]<-c(HR,

HR.true,

n.interim,

n.total,

n.interim,

n.interim,

n.interim,

ssr.stop[8],

ssr.stop[8],

ssr.stop[8],

ssr.stop[10],

ssr.stop[11],

1-as.numeric(ssr.stop[11]),

1.00,

ssr.stop[11],

Pow2)

} else {

#Find sample size for SSR-increase using optimal total maximum number of events

ssr<-Exp_events(n.total, HR.true, Rec_rate, times, lambda.ctrl, sided.test, alph, Pow2, n.interim, n.total, n.increase.1, max.subj, simulation.sim, seed.sim)

res_vec_2[count, 1]<-HR

res_vec_2[count, 2:15]<-ssr

res_vec_2[count, 16] <- Pow2

}

#Simulate trial for pre-planned minimum number of events (with sample size decrease at IA)

ssr.A<-Exp_events((n.interim+1), HR.optimise, Rec_rate, times, lambda.ctrl, sided.test, alph, Pow2, n.interim, n.total, n.increase.2, max.subj, simulation.sim, seed.sim)

if (ssr.A[14]>Pow2){

#If conditional power is larger than target, use pre-planned minimum number of events

res.opt<- n.interim+1

} else {

#If conditional power is not larger than target

#Find optimal total maximum number of events, allowing SSR to increase or decrease

res <- optimize(function(x, HR.optimise, Rec_rate, times, lambda.ctrl, sided.test, alph, Pow2, n.interim, n.total, n.increase.2, max.subj, target, simulation.opt, seed.opt)

objfun(x, HR.optimise, Rec_rate, times, lambda.ctrl, sided.test, alph, Pow2, n.interim, n.total, n.increase.2, max.subj, target, simulation.opt, seed.opt),

interval = c(n.interim+1, Max.EVENTS), HR.optimise, Rec_rate, times, lambda.ctrl, sided.test, alph, Pow2, n.interim, n.total, n.increase.2, max.subj, target, simulation.opt, seed.opt)

res.opt<-ceiling(res$minimum)

#Find sample size for SSR-both using optimal total maximum number of events

ssr.A<-Exp_events(res.opt, HR.optimise, Rec_rate, times, lambda.ctrl, sided.test, alph, Pow2, n.interim, n.total, n.increase.2, max.subj, simulation.sim, seed.sim)

}

if (res.opt>Max.EVENTS | ssr.A[14]<(Pow2-0.02)){

#If conditional power using optimal total maximum number of events does not reach desired power, stop trial at IA for futility

ssr.stop<-Exp_events((n.interim+1), HR.true, Rec_rate, times, lambda.ctrl, sided.test, alph, Pow2, n.interim, n.total, 1, max.subj, simulation.sim, seed.sim)

res_vec_2A[count, 1:16]<-c(HR,

HR.true,

n.interim,

n.total,

n.interim,

n.interim,

n.interim,

ssr.stop[8],

ssr.stop[8],

ssr.stop[8],

ssr.stop[10],

ssr.stop[11],

1-as.numeric(ssr.stop[11]),

1.00,

ssr.stop[11],

Pow2)

} else {

#Find sample size for SSR-both using optimal total maximum number of events

ssr.A<-Exp_events(res.opt, HR.true, Rec_rate, times, lambda.ctrl, sided.test, alph, Pow2,

n.interim, n.total, n.increase.2, max.subj, simulation.sim, seed.sim)

res_vec_2A[count, 1]<-HR

res_vec_2A[count, 2:15]<-ssr.A

res_vec_2A[count, 16] <- Pow2

}

}

}

}

#Null scenario

for (Pow2 in Pow_vec) {

if (Pow2==0.8){

int=Opt_IA[1, 1]

} else {

int=Opt_IA[1, 2]

}

target=Pow2

Pow1=Pow2

HR.true=1

HR.optimise=0.68

for (HR in HR_vec){

print(HR)

count<-count+1

design <- getDesignGroupSequential(sided=sided.test, alpha=alph, beta=1-Pow1,

informationRates = c(int, 1),

typeOfDesign = "asOF")

ss.gsd <- getSampleSizeSurvival(design = design,

piecewiseSurvivalTime = times,

lambda2 = lambda.ctrl,

hazardRatio = HR,

accrualTime = 0,

accrualIntensity = Rec_rate,

followUpTime = 1)

#Find interim SS and min total SS

(interim.subj<-ceiling(ss.gsd$numberOfSubjects[1]))

(max.subj<-ceiling(ss.gsd$maxNumberOfSubjects))

#Find min total events and interim events

(n.total <- ceiling(ss.gsd$eventsPerStage[2]))

(n.interim <- ceiling(ss.gsd$eventsPerStage[1]))

n.increase.1<-n.total-n.interim #Only allow number of events to increase at IA

n.increase.2<-1 #Allow number of events to also decrease at IA

#Maximum possible number of events allowed to increase to (double number of events from fixed RCT)

Max.EVENTS<-2*SS.fix_vec$Max.events[SS.fix_vec$HR==HR & SS.fix_vec$Power==Pow2]

#Simulate trial for pre-planned minimum number of events (with no sample size recalculation)

ssr<-Exp_events(n.total, HR.optimise, Rec_rate, times, lambda.ctrl, sided.test, alph, Pow2, n.interim, n.total, n.increase.1, max.subj, simulation.sim, seed.sim)

if (ssr[14]>Pow2){

#If conditional power is larger than target, use pre-planned minimum number of events

res.opt<- n.total

} else {

#If conditional power is not larger than target

#Find optimal total maximum number of events, allowing SSR to increase only

res <- optimize(function(x, HR.optimise, Rec_rate, times, lambda.ctrl, sided.test, alph, Pow2, n.interim, n.total, n.increase.1, max.subj, target, simulation.opt, seed.opt)

objfun(x, HR.optimise, Rec_rate, times, lambda.ctrl, sided.test, alph, Pow2, n.interim, n.total, n.increase.1, max.subj, target, simulation.opt, seed.opt),

interval = c(n.total+1, Max.EVENTS), HR.optimise, Rec_rate, times, lambda.ctrl, sided.test, alph, Pow2, n.interim, n.total, n.increase.1, max.subj, target, simulation.opt, seed.opt)

res.opt<-ceiling(res$minimum)

}

if (res.opt>Max.EVENTS | ssr[14]<(Pow2-0.02)){

#If conditional power using optimal total maximum number of events does not reach desired power, stop trial at IA for futility

ssr.stop<-Exp_events((n.interim+1), HR.true, Rec_rate, times, lambda.ctrl, sided.test, alph, Pow2, n.interim, n.total, 1, max.subj, simulation.sim, seed.sim)

res_vec_2[count, 1:16]<-c(HR,

HR.true,

n.interim,

n.total,

n.interim,

n.interim,

n.interim,

ssr.stop[8],

ssr.stop[8],

ssr.stop[8],

ssr.stop[10],

ssr.stop[11],

1-as.numeric(ssr.stop[11]),

1.00,

ssr.stop[11],

Pow2)

} else {

#Find sample size for SSR-increase using optimal total maximum number of events

ssr<-Exp_events(res.opt, HR.true, Rec_rate, times, lambda.ctrl, sided.test, alph, Pow2,

n.interim, n.total, n.increase.1, max.subj, simulation.sim, seed.sim)

res_vec_2[count, 1]<-HR

res_vec_2[count, 2:15]<-ssr

res_vec_2[count, 16] <- Pow2

}

#Simulate trial for pre-planned minimum number of events (with sample size decrease at IA)

ssr.A<-Exp_events((n.interim+1), HR.optimise, Rec_rate, times, lambda.ctrl, sided.test, alph, Pow2, n.interim, n.total, n.increase.2, max.subj, simulation.sim, seed.sim)

if (ssr.A[14]>Pow2){

#If conditional power is larger than target, use pre-planned minimum number of events

res.opt<- n.interim+1

} else {

#If conditional power is not larger than target

#Find optimal total maximum number of events, allowing SSR to increase or decrease

res <- optimize(function(x, HR.optimise, Rec_rate, times, lambda.ctrl, sided.test, alph, Pow2, n.interim, n.total, n.increase.2, max.subj, target, simulation.opt, seed.opt)

objfun(x, HR.optimise, Rec_rate, times, lambda.ctrl, sided.test, alph, Pow2, n.interim, n.total, n.increase.2, max.subj, target, simulation.opt, seed.opt),

interval = c(n.interim+1, Max.EVENTS), HR.optimise, Rec_rate, times, lambda.ctrl, sided.test, alph, Pow2, n.interim, n.total, n.increase.2, max.subj, target, simulation.opt, seed.opt)

res.opt<-ceiling(res$minimum)

}

if (res.opt>Max.EVENTS | ssr.A[14]<(Pow2-0.02)){

#If conditional power using optimal total maximum number of events does not reach desired power, stop trial at IA for futility

ssr.stop<-Exp_events((n.interim+1), HR.true, Rec_rate, times, lambda.ctrl, sided.test, alph, Pow2, n.interim, n.total, 1, max.subj, simulation.sim, seed.sim)

res_vec_2A[count, 1:16]<-c(HR,

HR.true,

n.interim,

n.total,

n.interim,

n.interim,

n.interim,

ssr.stop[8],

ssr.stop[8],

ssr.stop[8],

ssr.stop[10],

ssr.stop[11],

1-as.numeric(ssr.stop[11]),

1.00,

ssr.stop[11],

Pow2)

} else {

#Find sample size for SSR-both using optimal total maximum number of events

ssr.A<-Exp_events(res.opt, HR.true, Rec_rate, times, lambda.ctrl, sided.test, alph, Pow2, n.interim, n.total, n.increase.2, max.subj, simulation.sim, seed.sim)

res_vec_2A[count, 1]<-HR

res_vec_2A[count, 2:15]<-ssr.A

res_vec_2A[count, 16] <- Pow2

}

}

}

colnames(res_vec_2)<-c("Prior.HR",

"True.HR",

"n.interim",

"n.total",

"n.max",

"E.events",

"Max.events",

"E.subjects",

"Int.subjects",

"Max.subjects",

"E.SD",

"Prob.stop.Eff",

"Prob.stop.Fut",

"Prob.stop",

"Power",

"Target.Power")

colnames(res_vec_2A)<-c("Prior.HR",

"True.HR",

"n.interim",

"n.total",

"n.max",

"E.events",

"Max.events",

"E.subjects",

"Int.subjects",

"Max.subjects",

"E.SD",

"Prob.stop.Eff",

"Prob.stop.Fut",

"Prob.stop",

"Power",

"Target.Power")
